# Measuring criticality in control of complex biological networks

**DOI:** 10.1038/s41540-024-00333-9

**Published:** 2024-01-20

**Authors:** Wataru Someya, Tatsuya Akutsu, Jean-Marc Schwartz, Jose C. Nacher

**Affiliations:** 1 Department of Information Science, Faculty of Science, Toho University, Funabashi, Chiba 274-8510 Japan; 2 Bioinformatics Center, Institute for Chemical Research, Kyoto University, Kyoto, Uji 611-0011 Japan; 3 School of Biological Sciences, University of Manchester, Manchester, M13 9PT UK

**Keywords:** Computational biology and bioinformatics, Mathematics and computing, Systems biology

## Abstract

Recent controllability analyses have demonstrated that driver nodes tend to be associated to genes related to important biological functions as well as human diseases. While researchers have focused on identifying critical nodes, intermittent nodes have received much less attention. Here, we propose a new efficient algorithm based on the Hamming distance for computing the importance of intermittent nodes using a Minimum Dominating Set (MDS)-based control model. We refer to this metric as criticality. The application of the proposed algorithm to compute criticality under the MDS control framework allows us to unveil the biological importance and roles of the intermittent nodes in different network systems, from cellular level such as signaling pathways and cell-cell interactions such as cytokine networks, to the complete nervous system of the nematode worm *C. elegans*. Taken together, the developed computational tools may open new avenues for investigating the role of intermittent nodes in many biological systems of interest in the context of network control.

## Introduction

Controllability analysis integrates control theory concepts with complex networks techniques^1,2^. Recent works have shown that minimum driver nodes (controllers) are not only important to control the network but also that they have been associated to important biological functions^3–7^. In particular, network controllability has been applied to identify cancer-related genes^3,8–12^, drug-targets^8,13^, disease-related non-coding RNAs (ncRNAs)^14^, virus-targeted proteins^3,15,16^ and has also been used to predict specific neuron functions^17^ as well as to unveil the human brain functional structure^18^.

Several network controllability methodologies, such as Maximum Matching methodology (MM)^1^, Minimum Dominating Set (MDS) methodology^2^, and Feedback Vertex Set (FVS) methodology^19^ have been proposed to determine the minimum set of driver nodes required to drive a system from an initial state to desired final state in finite time. However, the minimum driver node set is not necessarily determined uniquely in general. Therefore, we have to classify nodes into critical (a node that appears in all minimum driver node sets), redundant (a node that does not appear in any minimum driver node set) and intermittent (the remaining one). Note that this classification has been done in all major controllability approaches shown above^20–22^. Extensive data analyses have demonstrated that the critical nodes play a prominent role in protein interaction networks as well as in metabolic pathways based on identified associations between them and human diseases and other important biological functions in a cell^5–7,9,10,14–16,20,21^. Note that the analyses have not been limited to human cells, and that, for example, critical proteins have been observed as enriched among significantly overexpressed genes that are abundant in the spiral form of *Helicobacter pylori*, and that, therefore, they play a role in the mechanistic switch of the two bacterial forms^7^. In sharp contrast, redundant and intermittent control categories have received much less attention. While redundant nodes are not involved in control at all, intermittent nodes show a quite different profile because they are partially involved in control, and consequently their biological importance remains largely unexplored.

To quantify the likelihood that a node is a driver node in the context of a maximum matching approach, a so-called control capacity metric was proposed^23^. The solution was obtained by using a random sampling algorithm, which was used to obtain the distribution of control capacity in several real-world networks^23^. Here, we propose a criticality metric in the context of a Minimum Dominating Set (MDS) model that measures the importance of each intermittent node based on the number of times it appears in an MDS solution.

For an undirected graph $G\left(V,E\right)$, a subset *U* of *V* is called a dominating set (DS) if each node is in *U* or has at least a neighbor in *U*. The minimum dominating set (MDS) is a dominating set with the minimum cardinality. Note that MDS may not be determined uniquely. It was proven in ref. ^2^ that if every edge is bidirectional and every node in an MDS (resp., DS) can assign an arbitrary value to itself and send arbitrary values to all of its outgoing links separately, at any time point, then the system can be driven to any desired state by using MDS nodes (resp., DS nodes) as driver nodes, in the sense of linear structural controllability (in ref. ^24^). Furthermore, it was proven in ref. ^16^ that the system can be driven to any desired state by using MDS nodes as driver nodes for a certain class of non-linear discrete time systems, where similar properties may hold for wider classes of non-linear systems. For directed graphs, the same properties hold if we modify the definition of DS so that *U* is called a dominating set if each node is in *U* or has at least one incoming edge from some node in *U*.

**Fig. 1:**
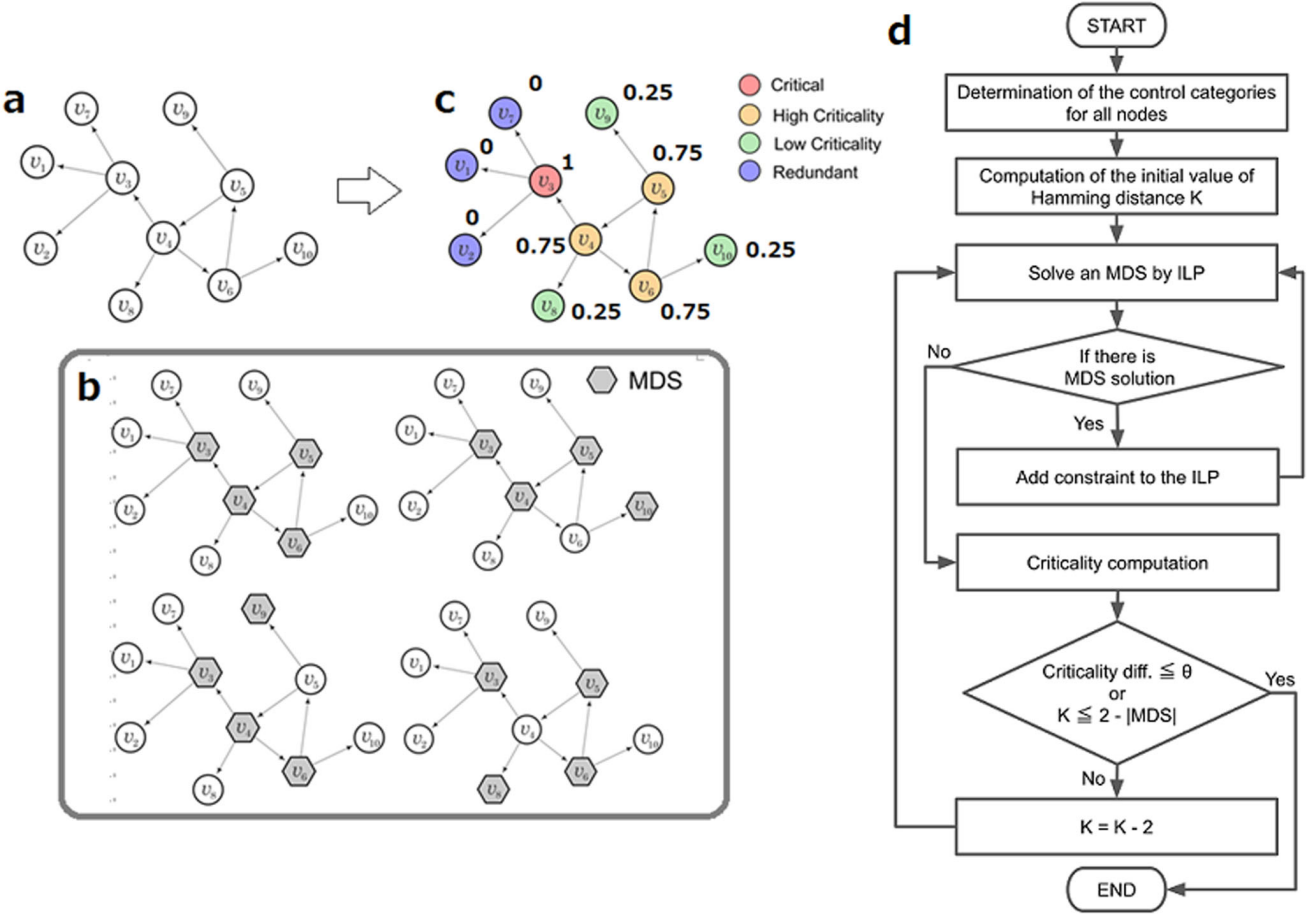
Illustration of the criticality metric computation in a graph. **a** A directed graph composed of ten nodes. **b** The computation of the MDS leads to four configurations or solutions with the same MDS size |MDS | = 4. **c** The criticality score from Eq. 1 is computed and displayed next to each node (Fig. 1c). The nodes ${v}_{4},{v}_{5},{v}_{6},{v}_{8},{v}_{9},{v}_{10}$ are intermittent nodes, but nodes ${v}_{4},{v}_{5},{v}_{6}$ participate in three out of four possible solutions, therefore their criticality score is 0.75, showing higher importance than nodes ${v}_{8},{v}_{9},{v}_{10}$ (criticality score 0.25). Nodes with criticality score above 0.5 (below 0.5) are defined as high (low) criticality nodes, respectively. **d** Schema of the proposed algorithm for the criticality computation.

While the maximum matching problem can be solved in polynomial time, the MDS problem is a well-known NP-hard problem. That means it is not expected to find an algorithm that solves this problem in polynomial time. Therefore, when the size of network increases, its computational time increases exponentially. Because the computation of criticality not only requires the computation of a single MDS solution but requires to know all possible solutions, it is clear that the criticality metric, although simple in its definition, poses significant computational challenges. Because of the intrinsic complexity of this problem, and to efficiently compute criticality metric, we developed a new algorithm (see Fig. 1d and details in the Methods section) and applied it to several biological networks to identify intermittent nodes with high criticality associated with relevant bio-medical functions. It is worth noting that while the sampling method proposed in^23^ may be used to estimate the criticality based on MDS, their method is based on Markov chain and thus convergence to the uniform distribution may need many repetitions. Since MDS is NP-hard and thus its computation needs long CPU time (different from maximum matching), it is preferable to use a small number of repetitions. Therefore, we developed another heuristic method to estimate the criticality based on MDS.

Suppose that we have a directed graph $G\left(V,E\right)$ where $V=\left\{{v}_{1},{v}_{2},\ldots ,{v}_{N}\right\}$ is a set of nodes and *E* is a set of directed links. Let *M*_*set*_ be the set of all the minimum dominating sets (MDSs) as its elements for the $G\left(V,E\right)$, and M be an element of this set. We then define the criticality of each *v*_*i*_ by

1 $${{CR}}_{i}=\frac{\left|\left\{M\in {M}_{{set}}{\rm{|}}{v}_{i}\in M\right\}\right|}{\left|{M}_{{set}}\right|}$$

Note that this definition can also be applied to undirected graphs by considering a graph *G* with a set *E* of undirected links.

**Fig. 2:**
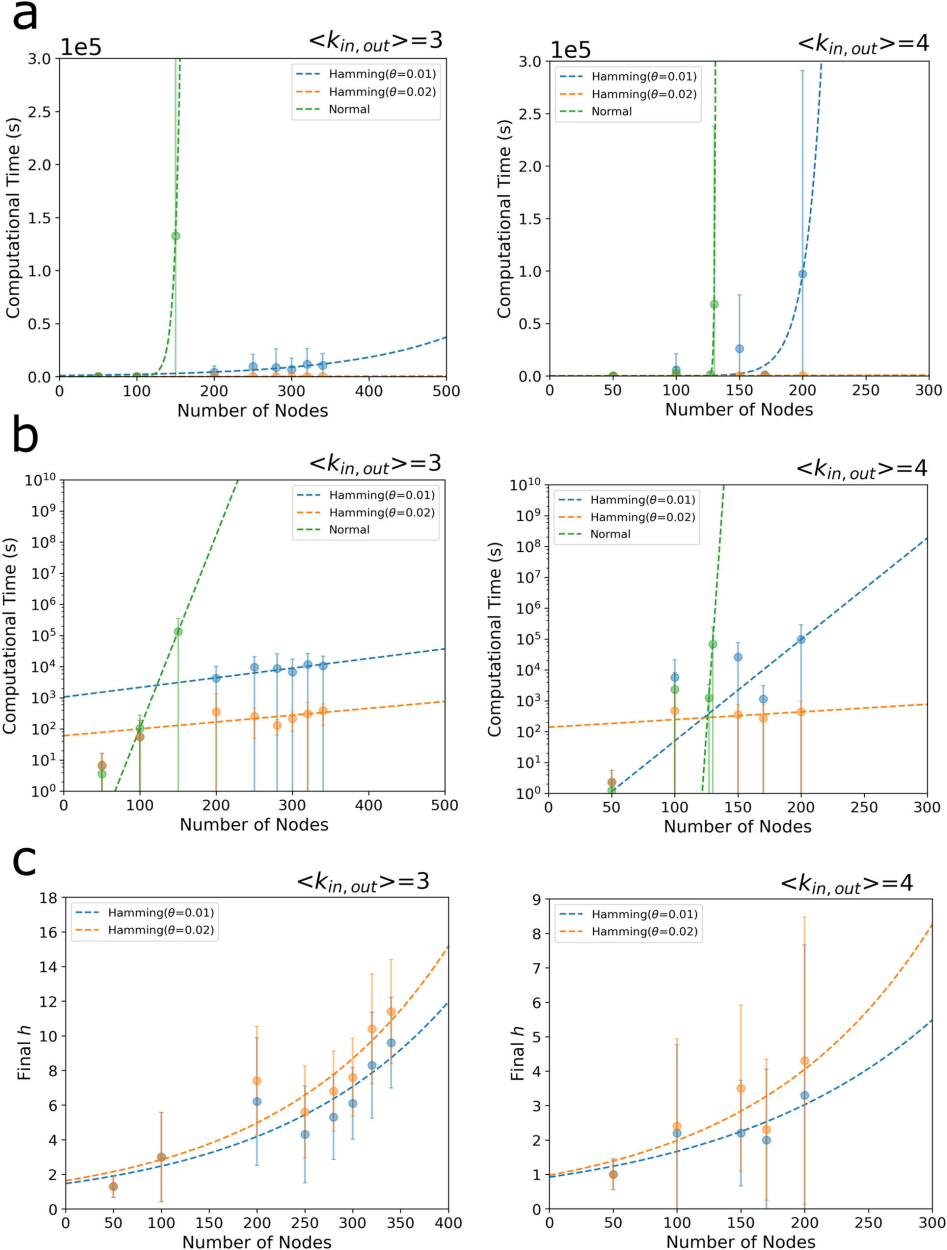
Computational time versus the network size. **a** The computational time in linear scale. **b** The computational time in log scale. By using the Hamming distance with Hamming parameters $\theta =0.01$ and $\theta =0.02$ significant improvements are observed. **c** The dependence between the final *h* value and the network size is shown. In each figure, the computation was repeated and averaged over ten network samples. The mean value of the computation time is shown as a dot and the standard deviation as an error bar.

By using the metric shown in Eq. 1, we developed and implemented an efficient algorithm to compute criticality in directed biological networks. The proposed algorithm uses the Hamming distance concept among others to make feasible the computation in large networks (see Fig. 1d). As described above, and shown in Eq. 1, the criticality calculation is computationally intensive since it requires all possible MDS solutions, which increases exponentially with the network size. Moreover, the MDS problem itself is an NP-hard problem. Therefore, new efficient algorithms are required for its computation. First, we proposed and implemented an algorithm to efficiently computer criticality. The performance of the algorithm was evaluated using artificially generated scale-free networks (see Fig. 2 and Supplementary Figs. 1–5). The computational experiments demonstrated the correctness of the solution of the proposed heuristic algorithm as well as the improvements obtained in the computational time by using Hamming distance techniques among others. Next, we applied the proposed criticality algorithm to three biological networks and systems: the human RTK signal transduction pathway, the human cytokine-cytokine interaction network and the connectome of the nematode *Caenorhadbitis elegans*. The application of the developed algorithm to several biological networks unveiled that the intermittent nodes play a more important role than previously assumed. First, our findings show that the set of high criticality proteins in the human RTK signalling pathway are associated with phosphorylation and cancer. Moreover, they are statistically significantly enriched with disease genes associated to 16 specific human disorders, from congenital abnormalities to musculoskeletal diseases. Second, by taking advantage of the latest development on assembling cell-cell interaction data as well as cytokine-cytokine interaction network, we performed a criticality analysis of these networks with special attention of the inflammation network module related to the COVID-19 patients. While for the cell network most of the critical cells are involved or adjacent to this inflammation module, the results for the cytokine network show that the algorithm does not only recover well-known cytokines that are part of the module such as IL4 (critical) and CSF2 (high criticality), but also identifies new ones that are adjacent to this module such as IL20 (critical) and IL7, IL18 and TNFSF13 (high criticality), suggesting similar functional roles. The analysis of the neural network of *C. elegans* organism also assisted to classify intermittent nodes with high criticality into specific neuron types. Taken together, the application of the proposed criticality analysis in biological systems and the developed algorithmic tools in the context of controllability theory makes it possible to unveil previously overlooked biological functions of intermittent nodes in large networks.

## Results

In this study, we first evaluated the performance of the algorithm using artificially generated scale-free networks. We then applied the proposed efficient criticality algorithm to the signal transduction pathways, cell-cell and cytokine-cytokine interaction networks and the connectome of the *C. elegans* organism.

### Overview of the proposed criticality algorithm in MDS

Here we summarize the main computational steps involved in the proposed algorithm before showing the data analysis results. Note that the algorithm is described in detail in the Methods section and that the proofs of two mathematical propositions derived by us and involved in the algorithm can be found in the Methods section. As shown in the scheme (Fig. 1d), the first step requires the identification of all nodes control categories in the MDS. This can be efficiently computed by a direct application of the algorithm and techniques described in^21^. Next, the algorithm computes the initial value of Hamming distance *K* (between an existing MDS solution and the newly identified MDS solution) and calculates an MDS by using Integer Linear Programming (ILP) under the constraint that the Hamming distance must be greater or equal than *K* (see Eq. 20, the last constraint in the Methods section). The algorithm repeats the process until no solution for MDS is found (see Fig. 1d). Then, the criticality metric (Eq. 1) is computed. If the difference of criticality between two rounds is enough small (less than a threshold *θ*, where *θ* is a parameter that determines the precision of the computed criticality (see Supplementary Figs. 1–5)) or $K\le 2-{|MDS|}$, then the algorithm terminates. Otherwise, it updates the Hamming distance by reducing the value as K = K-2. Then, the algorithm re-starts the computation with different Hamming distance (see Fig. 1d). Note that the *θ* parameter can be adjusted in order to improve the accuracy of the criticality computation. In this work, experiments were done with small value of $\theta =0.01,\theta =0.02$ (see red lines in Supplementary Figs. 1–5).

### Algorithm performance using artificially generated scale-free networks

First, to assess the computational performance of the algorithm using the Hamming distance, the computation time was measured using artificially generated scale-free networks. The degree exponent γ of the networks used in the analysis is 2.5. The results for average degrees $< {k}_{{in},{out}} > =\mathrm{3,4}$ are shown in Fig. 2a, b. The horizontal axis represents the number of nodes in the network and the vertical axis represents the computation time (seconds). The Hamming ($\theta =0.01,\theta =0.02$) curves are the results of the criticality algorithm using the Hamming distance with the specified value *θ* of 0.01 and 0.02 to terminate the calculation, respectively (see Fig. 2). The green dashed-line (normal) curve is the result when the criticality is calculated without using the Hamming distance-based algorithm. The results shown in Fig. 2a, b indicate that the proposed algorithm with Hamming distance provides significant improvements in computational time.

To assess the effect of the average degree, we consider two cases for $< {k}_{{in},{out}} > =3$ and 4. As shown in Fig. 2a, b (left and right), there is a shift in the Hamming ($\theta =0.01$) curve as the network is more complex for higher average degree, but the shift is very large indicating that the computation is significantly influenced by the average degree. In addition, the standard deviation is relatively large for the same network size, indicating that the criticality computation problem is even more complex than that of the original MDS which is a well-known NP-hard problem.

The Hamming distance *K* for the ILP calculation at the end of the computation is expressed as $K=2h-\left|{MDS}\right|$ (here, *h* means the number of the different nodes between an MDS sample and the new one), and from this, the value of *h* reads as $h=(K+\left|{MDS}\right|)/2.$

Note that we use a capital letter *K* to denote the Hamming distance, whereas we use lower-case letters, *k*_*in*_, *k*_*out*_ and *k* to denote the in-degree, out-degree, and total degree of a node, respectively.

We then computed the dependence between the final *h* value and the network size, as shown in Fig. 2c. While for $< {k}_{{in},{out}} >$ = 3, the *h* value grows fast and becomes larger when increasing the network size, the curve displays a lower slope and takes smaller values for $< {k}_{{in},{out}} > =4$, being around 1 ~ 3 even with large increments of network size. By comparing Hamming curves $(\theta =0.01$) and ($\theta =0.02$) with $< {k}_{{in},{out}} > =4$, we observe that the h at the end of the calculation only differs by 1 ~ 2 as an average, but when combined with the information from Fig. 2a, b, the computational times becomes larger even when the h is different only by 1 or 2, because the number of MDS solutions with the same size also rapidly increases. Additional analyses on the algorithm performance and accuracy can be found in Supplementary Note 1 and Supplementary Figs. 1–5.

### High criticality proteins in RTK signalling pathways are associated with phosphorylation and cancer

The Receptor Tyrosine Kinases (RTKs) pathway is the largest subnetwork of the signal transduction pathways and plays a key role in many biological processes such as growth, cell differentiation and metabolism^25^. However, down-regulation and alterations in its genes lead to multiple human diseases, including various types of cancer. The signalling pathways have recently been analysed using controllability methods such as maximum matching^8^, minimum dominating sets^21^, minimum feedback vertex set^22^. While in all these studies the proteins of the pathway can be classified into three control categories, namely, critical, intermittent and redundant, the relative importance of the intermittent nodes in the network may have been overlooked.

**Fig. 3:**
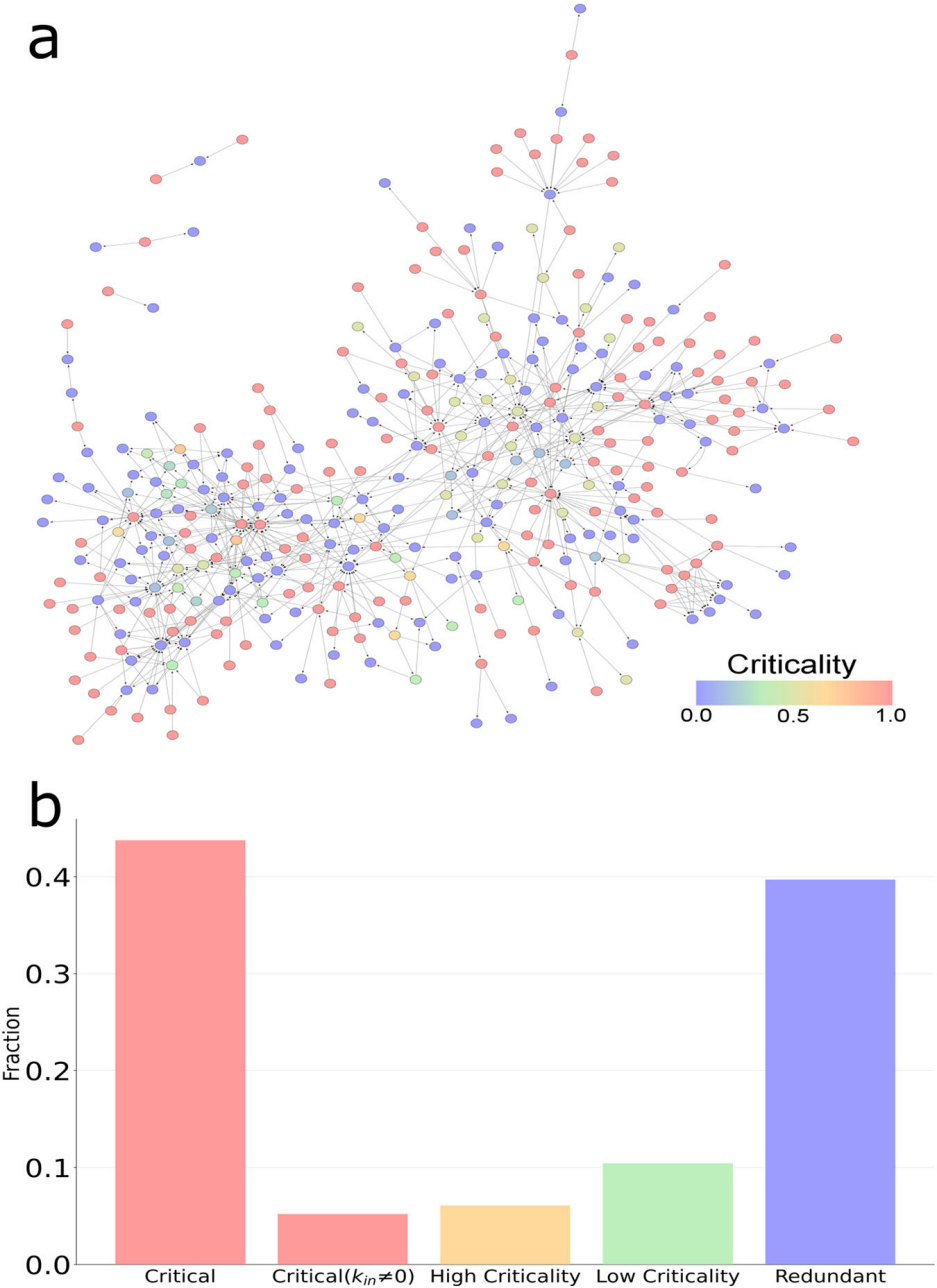
Criticality in the RTK signalling pathway. **a** The RTK signalling pathway with the control categories mapped onto each node. **b** The fraction of the control categories in the RTK signalling pathway. Nodes with criticality value above (below) 0.5 are classified as high (low) criticality.

First, we mapped the computed criticality metric onto the RTK signalling pathway (Fig. 3a). This continuous measure contrasts with previous discrete definitions of control classifications. Interestingly, most of the intermittent nodes have a large range of values of criticality and a noticeable fraction of them also has a high criticality above 0.5 (Fig. 3b). A detailed list of the proteins with high criticality is shown in Supplementary Table 1 (Excel file). The network also contains a large fraction of critical nodes. The reason is that many proteins act as receptors without incoming links from other proteins. The fraction of critical nodes excluding receptors (in-degree k_in_≠0) is shown on the figure.

We then used a set of phosphorylated proteins derived from PhosphositePlus^8,26,27^ to determine if there was a relation between phosphorylation and high criticality. PhosphositePlus is a comprehensive, manually curated resource gathering information about experimentally observed post-translational modifications (PTM). Cell rapid responses to environmental changes and conditions are mediated by the modification of certain protein binding features which are orchestrated by PTMs such as acetylation, ubiquitination and phosphorylation (pS/pT and pY). In this analysis, the subset of proteins affected by tyrosine phosphorylation (pY) was used.

**Fig. 4:**
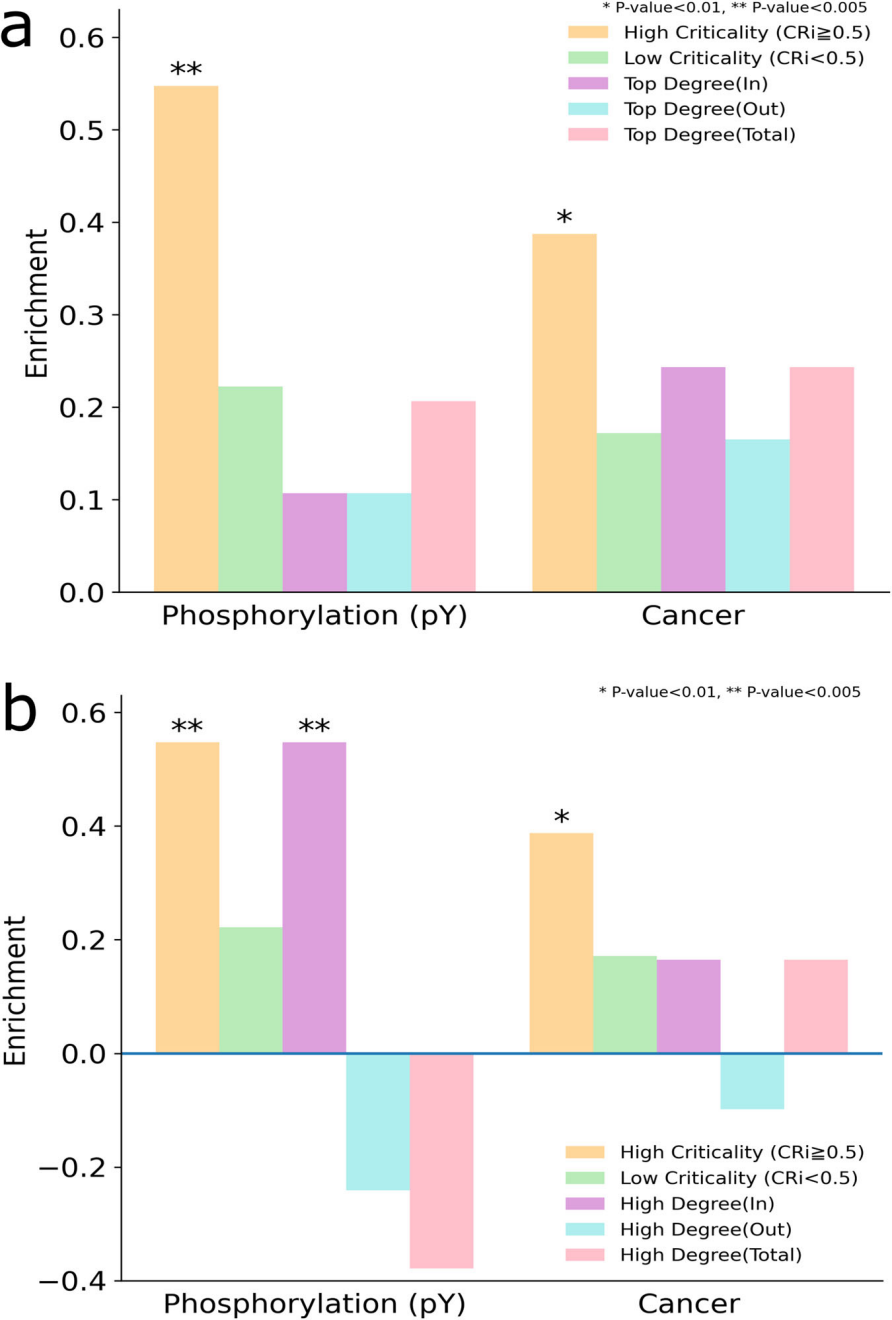
Enrichment of high and low criticality proteins of RTK signaling pathways with phosphorylation (pY) and cancer datasets. Enrichment for in-degree *k*_*in*_, out-degree *k*_*out*_, and total degree k are also shown for (**a**) the top degree and (**b**) high degree node configurations. All *p*-values derived from two-tailed Fisher’s exact tests.

The statistical analysis shows that the high criticality proteins are statistically significantly enriched with tyrosine phosphorylation (pY) mechanism (*p*-value = 0.0046; all p-values derived from two-tailed Fisher’s exact tests) (see Fig. 4). In contrast, the low criticality proteins are not significantly enriched by phosphorylation. This shows the biological importance of intermittent nodes which seems to be highly regulated by PTM related processes. See the Supplementary Note 2 and Supplementary Table 1 (Excel file) for the list of the high criticality proteins associated with the tyrosine phosphorylation (pY) mechanism.

In addition, we used a cancer-related gene-centric data from CancerGenes database which integrates four public resources namely, NCBI Entrez Gene, Ensembl BioMart, active promoter regions and the Sanger Institute COSMIC^8,28^. Then, we similarly mapped it on the RTK signaling pathway. The results also showed a statistically significant enrichment of high criticality proteins with cancer genes (*p*-value = 0.0059; two-tailed Fisher’s exact test) (see Fig. 4). See also the Supplementary Table 1 (Excel file) for the list of proteins with high criticality associated with the cancer genes set.

To assess the relation between high criticality and node degree, we recomputed the enrichment in the mentioned above biological datasets by using node degree as a metric instead of high criticality. In a directed network, a node has in-degree *k*_*in*_ and out-degree *k*_*out*_, and the sum of both is called total degree k. First, we considered a set of *top degree* nodes, which are obtained by selecting the same number of high criticality nodes as observed in the RTK signaling network ordered by degree. The second set is a set of *high degree* nodes composed by the same number of high criticality nodes as observed in the RTK signaling network and ordered by degree but excluding the same number of nodes as critical nodes observed in the network. Because our high criticality enrichment analysis is excluding the critical nodes (i.e., criticality score 1), this set also represents a fair benchmark for comparison purposes. Note that for each set, three sets are actually computed using in-degree *k*_*in*_, out-degree *k*_*out*_, and is total degree *k*. As shown in Fig. 4a, for top degree analysis, the high criticality gives highest enrichment and statistical significance for both phosphorylation and cancer genes sets.

For the high degree analysis, the results shown in Fig. 4b indicate that high criticality also gives higher enrichment than the rest of metrics, and only for *k*_*in*_ gives the same enrichment score. However, this case is not relevant for control purposes. Note that for *k*_*out*_, there is negative enrichment (depletion). Therefore, this analysis reveals the specificity of high criticality nodes in the investigated datasets.

### High criticality proteins in RTK signalling pathways are also associated with specific diseases

A closer insight on the high criticality proteins can be obtained by examining larger datasets corresponding to multiple and diverse human diseases. Here we used sets of genes corresponding to 299 diseases, which were used previously to unveil human disease modules in the interactome network^29^, and mapped them on the RTK signaling pathway.

**Fig. 5:**
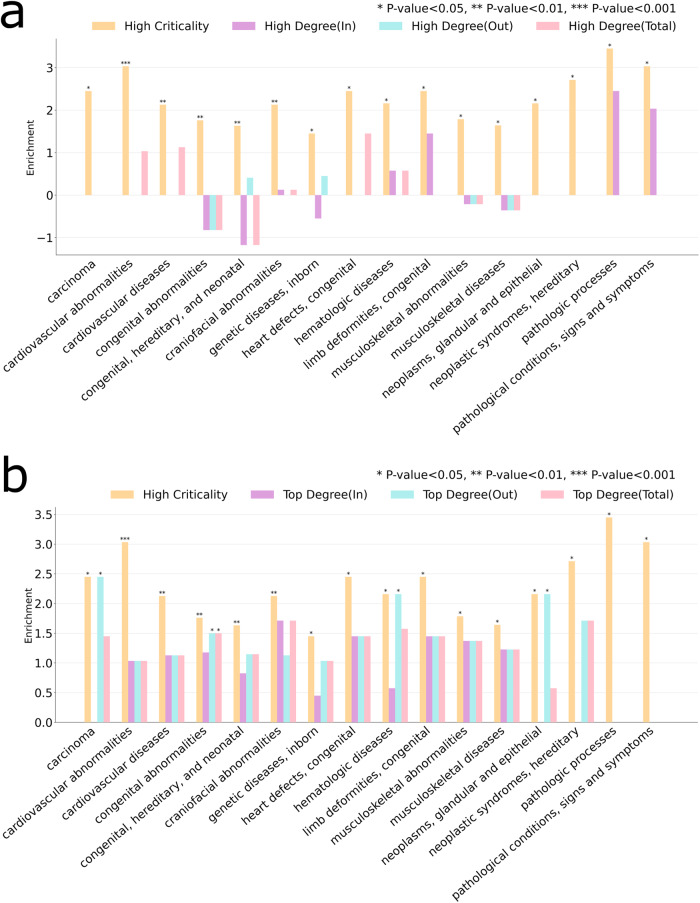
Enrichment of high criticality proteins in the RTK signaling pathways with human diseases. These diseases show a statistically significantly enrichment with high criticality. Enrichment for *k*_*in*_, *k*_*out*_ and total degree *k* are also shown for (**a**) the high degree and (**b**) top degree sets of nodes, respectively. All *p*-values derived from two-tailed Fisher’s exact tests.

As shown in Fig. 5, the statistically significantly enrichment of high criticality proteins is observed in 16 different diseases. For example, congenital, hereditary and neonatal diseases are enriched by high criticality proteins. See the Supplementary Table 1 (Excel file) for a list of the identified high criticality proteins associated with each disease. It has been reported that developmental abnormalities and syndromes are linked to both cancer and RTK signaling pathways^25,30^. Our algorithm identifies high criticality proteins that overlapped significantly with this disease (*p*-value = 0.0047; two-tailed Fisher’s exact test). Among cardiovascular diseases, the ischemic heart disease represents the largest cause of deaths in the world. It has also been reported that specific RTK targets are required to be regulated in gene expression to successfully treat ischemic heart diseases^31^. The analysis showed a statistically significant association of the cardiovascular disease (*p*-value = 0.0091; two-tailed Fisher’s exact test) and cardiovascular abnormalities (*p*-value = 0.0006; two-tailed Fisher’s exact test) with high criticality proteins. Several works have also highlighted the importance of inhibiting of RTKs targets as an improved therapeutic strategy for vascular disorders^32^. Moreover, we computed the fraction of critical, high criticality, low criticality, and redundant proteins in the RTK signaling pathway.

To assess that the high criticality measure outperforms degree centrality, we performed the same analysis as shown in Fig. 4 by using the high degree (Fig. 5a) and top degree (Fig. 5b) sets of nodes. The results clearly indicate that the identified set of high criticality proteins show the highest enrichment in all cases for high degree nodes, and also in almost all cases for top degree nodes except in three diseases for which *k*_*out*_ gave the same result.

**Fig. 6:**
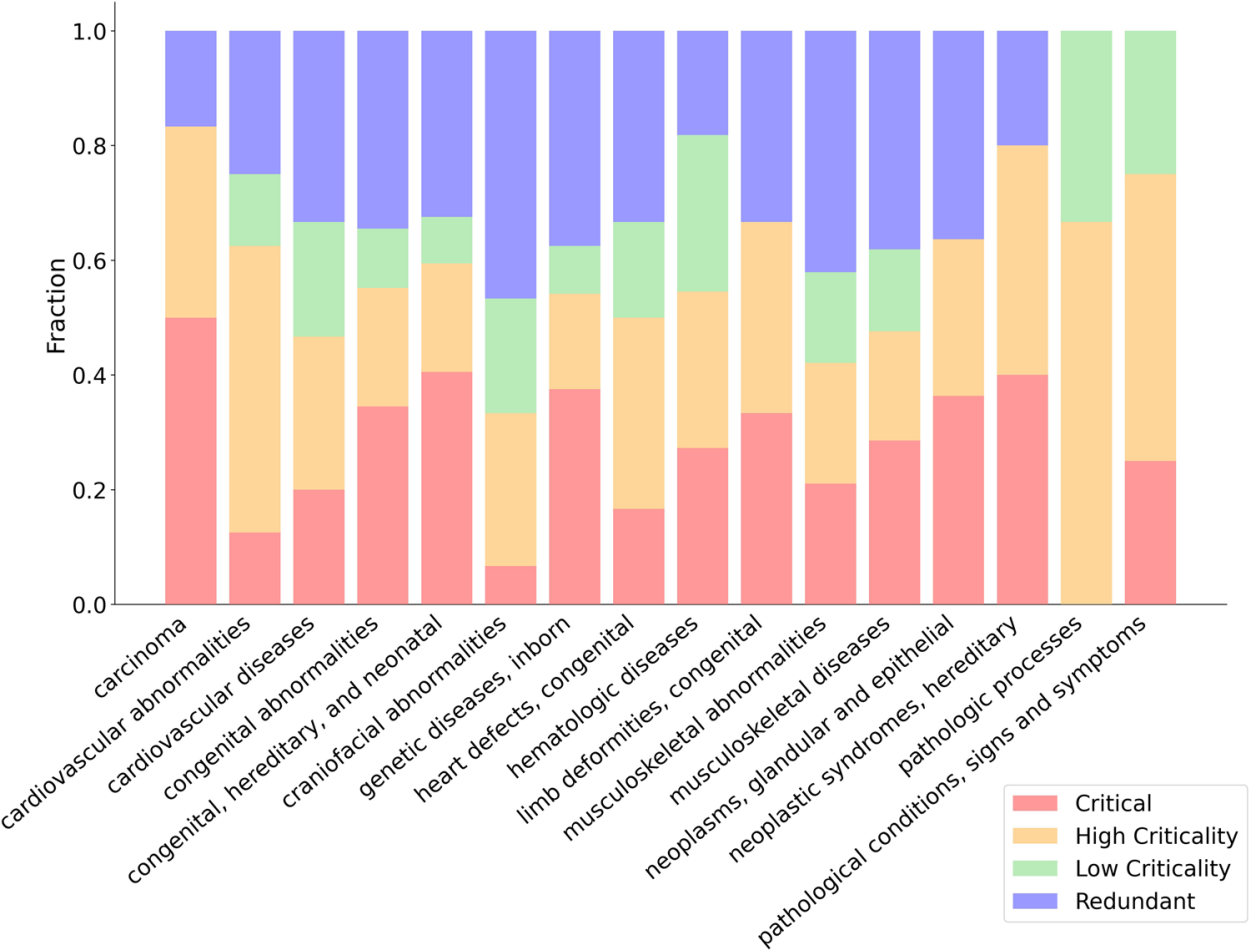
Fraction of control categories for each disease. The fraction of each control category (critical, high and low criticality and redundant) of proteins in the RTK signaling pathway and computed for each disease.

Figure 6 shows the fraction of critical and high criticality nodes computed for each disease. Critical nodes appear in all of the MDS solutions, therefore are regarded as important nodes for control. Interestingly, the high criticality fraction tends to be sometimes larger than the critical one. In almost all of the analysed diseases, the combination of critical nodes and high criticality nodes represents more than half of the total nodes. The importance of these intermittent nodes that appear in many MDS solutions could have not been unveiled without using our proposed algorithm. Pathological processes, for example, do not have critical proteins, but a large fraction of the nodes are high criticality proteins.

### Key cytokines computed from cell interactions data in Covid-19 patients have high criticality

A recent study has inferred cell-cell interactions and cytokine-cytokine interactions that can be used to investigate multiple biological and disease processes such as inflammatory diseases^33^. Each cell produces cytokines and different cells also have cytokine receptors. The combination of cytokine-receptor interactions and the tissue/cell-cytokine and receptor interactions led to obtaining two networks called cell-cell interaction network and cytokine-cytokine interaction network.

The cell network consists of 18 blood cell types and 24 tissues that are combined as 42 nodes. By means of the cytokines, a total of 581 interactions and communication channels between two cells were determined. The cytokine network consists of 115 nodes and the number of interactions between cytokines is 2818.

We then applied our criticality algorithm to both networks and determined the control categories of each network including critical, high and low criticality nodes.

**Fig. 7:**
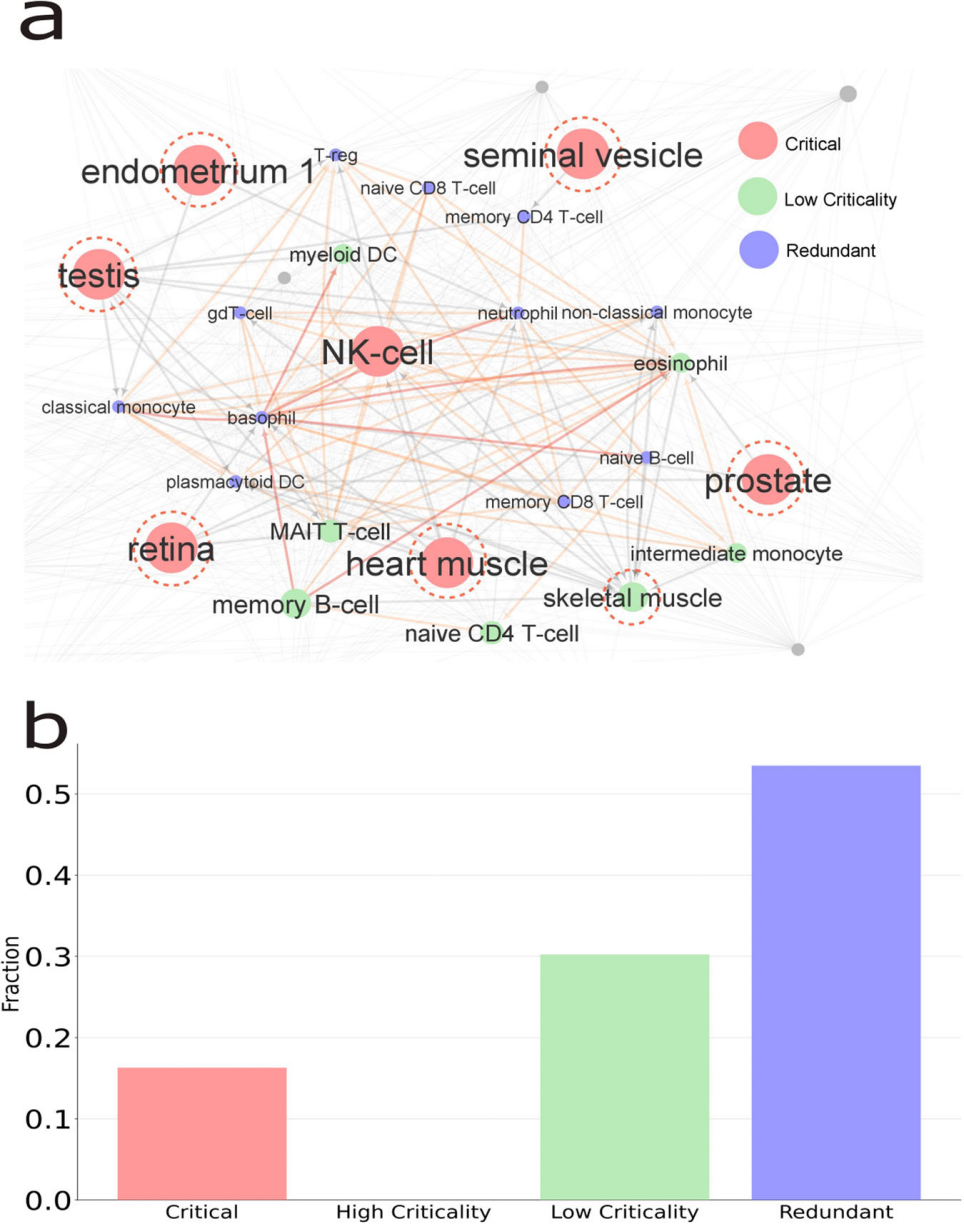
Criticality in the cell-cell interactions network. **a** The network of cell-cell interactions mediated by cytokine interactions elevated in COVID-19 patients (orange links). The red links represent interactions mediated by IL4 and IL5 cytokines, which are key cytokines involved in the inflammatory process. The figure shows the critical, low criticality and redundant cells in red, green and blue, respectively. Red cells with dashed-circles denote identified critical proteins adjacent to the cells of the COVID-19 module. **b** The fraction of control categories in the cell-cell interaction network.

Figure 7a shows a part of the entire cell network. The grey links correspond to the cell-cell interaction networks. The orange links correspond to those inter-cellular interactions involving elevated cytokines in COVID-19 patients^33^. The red links represent interactions mediated by IL4 and IL5 cytokines, which are key cytokines involved in the inflammatory process. The figure shows the critical, low criticality and redundant cells in red, green and blue, respectively. Interestingly, all the critical cells identified in our analysis were adjacent to at least another cell already involved in the highlighted module of COVID-19 (see the six red nodes with dashed circles) or already present in the module such as the NK-cell. This suggests that the critical cells may also play a key role in the COVID-19 inflammatory response. Moreover, the skeletal muscle cell type shows up as low criticality and it is also connected to the main module. Myeloid DC, memory B- cell, MAIT T-cell and intermediate monocyte belong to the module and are also identified as low criticality cells. NK-cell is a critical cell and it is also included in the COVID-19 module.

As shown in Fig. 7b the network has approximately a 30% of nodes classified as intermittent nodes with low criticality and 17% of nodes are critical nodes.

**Fig. 8:**
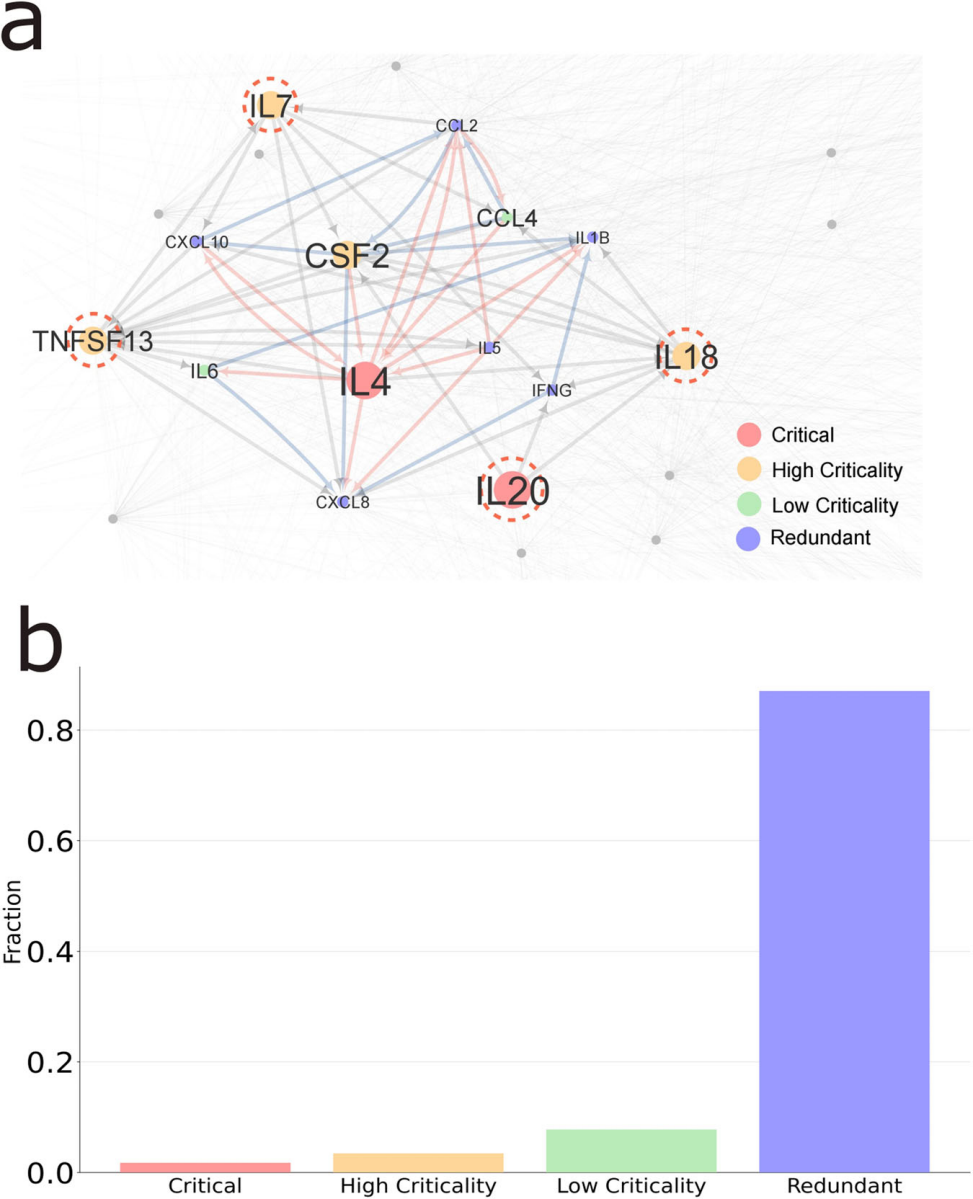
Criticality in the cytokine-cytokine network. **a** Fraction of the network of cytokine-cytokine interactions selected from those cytokines elevated in COVID-19 patients (blue links). Red links denote cytokine-cytokine interactions involving IL4 and IL5. Grey links denote the background links of the rest of the cytokine-cytokine network. Control categories are denoted by colors as shown in legend. **b** The fraction of control categories in the cytokine-cytokine interaction network.

The complete cytokine network consists of 115 nodes and 2818 interactions between cytokines^33^. Figure 8a shows the subnetwork for the cytokines interactions that is overexpressed (blue links) in COVID-19 patients. Red links denote cytokine-cytokine interactions involving IL4 and IL5 cytokines. This network module is enlarged by several critical and high critical cytokines identified by our proposed algorithm. Interestingly, the IL20 cytokine is a critical cytokine and it is adjacent to the main module. The IL7, IL18 and TNFSF13 are high criticality cytokines identified by the algorithm. They are also adjacent to the main module, therefore, they are denoted with dashed lines. Finally, the CSF2 is a central cytokine in the module that has also high criticality value. This analysis shows that the combination of critical, high and low criticality control categories may lead to expand the knowledge of specific disease modules.

The cytokine interaction network consists of a small fraction of critical nodes. One of the reasons for this is that the network is dense in terms of number of interactions among cytokines. Interestingly, this network shows a noticeable high criticality fraction which is even larger than that of the critical category (see Fig. 8b). This highlights the importance of the developed algorithm because it enables us to unveil control-related features in important subnetworks and molecules. Moreover, as shown in Fig. 8a these high criticality cytokines were not located randomly in the network. Instead, they were adjacent to at least one cytokine involved in the COVID inflammation network module. A list of the cytokines with the highest criticality values are shown in Table 1.

**Table 1:** Critical and high criticality cytokines in the COVID-19 cytokine-cytokine interaction network.

Cytokine	Category	Criticality
IL4	Critical	1.0
IL20	Critical	1.0
CSF2	High Criticality	0.75
IL18	High Criticality	0.75
IL7	High Criticality	0.625
TNFSF13	High Criticality	0.5

**Fig. 9:**
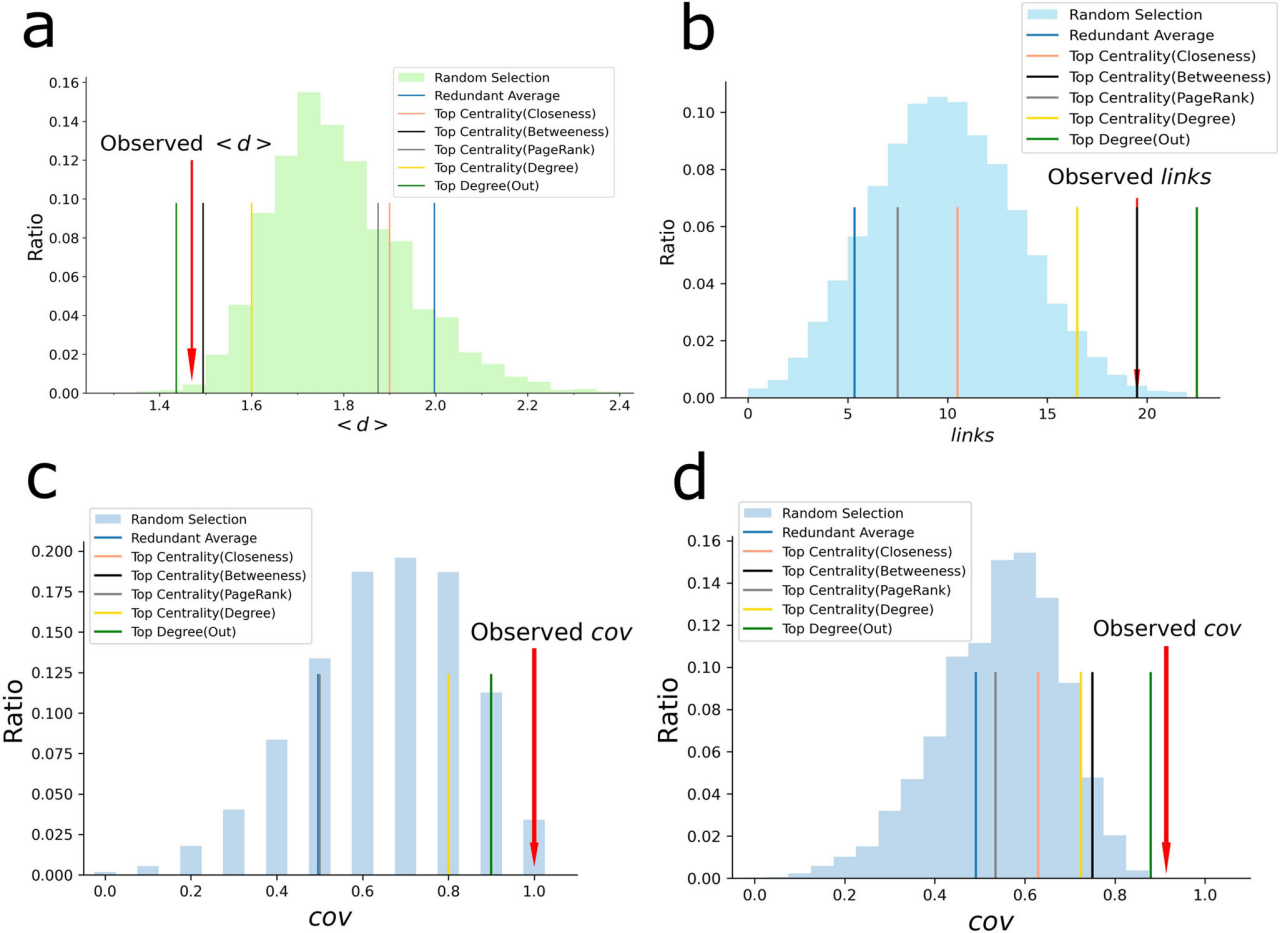
Network metrics analysis for high criticality nodes. **a**–**c** Histograms showing a comparison of <*d* > , *links* an*d cov* metrics computed for the COVID-19 module in the cytokine network for high criticality nodes (red arrows) and randomly selected nodes (histogram bars). **d** The high criticality nodes coverage for the entire cytokine interaction network. The number of trials of random selection for each feature is 10,000. Each figure also displays the results for the top degree/centrality set of nodes computed using closeness, betweenness, page rank, out-degree and total degree metrics for comparison purposes.

In order to verify that the high criticality nodes (the four nodes shown in yellow in Fig. 8a) identified in the cytokine interaction network can indeed have stronger control properties on the COVID inflammation module than randomly selected nodes, we computed a series of metrics, namely the average shortest path from high criticality nodes to the network module <d > , the total number of all directed edges outgoing from any high criticality node and incoming to any node in the module (*links*) and the fraction of nodes in the module that have an incoming edge from high criticality nodes (*cov*) (that is, the module coverage ratio by the high criticality nodes) (see methods section for mathematical definitions). In each case from Fig. 9a to Fig. 9d, the values of the metric for the four high criticality nodes are indicated by red arrows and the average metric value of the redundant nodes in the module for COVID-19 is indicated by a blue line for comparison. Each histogram shows the distribution of each metric computed by randomly selecting four nodes from the cytokine interaction network. The number of trials of random selection for each metric is 10,000. The vertical axis of each figure shows the frequency of occurrence of the metric values of the randomly selected nodes as a percentage, and the horizontal axis shows the metric value.

In Fig. 9a, the <*d*> value of the high criticality nodes is clearly smaller than the majority values obtained computed using randomly selected samples (*p*-value = 0.0063; one-tailed randomization test). This confirms that the high criticality nodes can control the module more easily than other nodes. In Fig. 9b, the *links* for the high criticality nodes are around 20 (*p*-value = 0.0045; one-tailed randomization test), while the *links* value for the randomly selected nodes peaks at around 10 in average, indicating that high criticality nodes have a greater impact on the module than the other nodes. In Fig. 9c, the *cov* metric of the module nodes is 1.0 for the high criticality nodes, indicating that these nodes are enough to effectively controlling the module (*p*-value = 0.0341; one-tailed randomization test). Note that Fig. 9c shows a discontinuous histogram, but this is because there are 10 module-constituting nodes and only 11 possible values for the coverage ratio. Figure 9d shows that the high criticality nodes coverage for the entire cytokine interaction network is over 90% (*p*-value = 0; one-tailed randomization test), indicating that the regulation of these cytokines may have a significant impact not only on the regulation of modules related to COVID-19, but also on other diseases.

To assess our results in comparison with other metrics, we selected five centrality metrics, namely, closeness, betweenness, page rank, out-degree and total degree. We then computed the same metrics <*d* > , *links*, and *cov* by using the set of top degree/centrality nodes (Fig. 9) and the set of high degree/centrality nodes (Supplementary Fig. 6 and Supplementary Note 3) ordered according to each centrality measure mentioned above. For the set of top degree/centrality nodes, the high criticality performs better than all the examined centrality for the *cov* metric in both the COVID module (Fig. 9c) and the entire network (Fig. 9d). Regarding <*d*> and *links* metrics, high criticality scores second after the out degree (Fig. 9a, b). Next, the results for the high degree set of nodes also show the best performance for the high criticality nodes when the *cov* metric is examined for the complete network (Supplementary Fig. 6d), the best result for the coverage of the COVID module scoring same as the out-degree (Supplementary Fig. 6c), and shows the second rank in the results for <*d*> and *links* measures (Supplementary Fig. 6a, b). It is worth mentioning that coverage is the metric most closely related to actual control among the other two. Moreover, it is to be noted that the high criticality nodes are part of a set that satisfies controllability properties derived by advanced techniques^2^, while sets obtained by other centrality metrics such as page rank or degree, although they can be used to rank network nodes, do not satisfy controllability conditions.

### Motor neurons in the *C. elegans* connectome tend to have high criticality

The nematode worm *C. elegans* is the only organism for which a detailed completed map of the nervous system has been reconstructed at the single neuron level resolution. It is therefore one of the best examples to test and apply control theory methods^17^. The *C. elegans* connectome is a highly dense directed network composed of several neuron classes. The network and neuron classification were compiled from information obtained from WormAtlas database^34,35^. Ambient stimuli induce responses of sensory neurons acting in a role of input signal nodes distributing the incoming signals through a network composed of 281 neurons and 97 muscles (nodes), being connected by a total of 5256 directed synaptic interactions and/or neuro-muscular junctions. Motor neurons are connected to muscles, while inter-neurons mediate between neurons distributing the signals. Poly-modal neurons refer to those neurons classified as capable of double or more functionality.

**Fig. 10:**
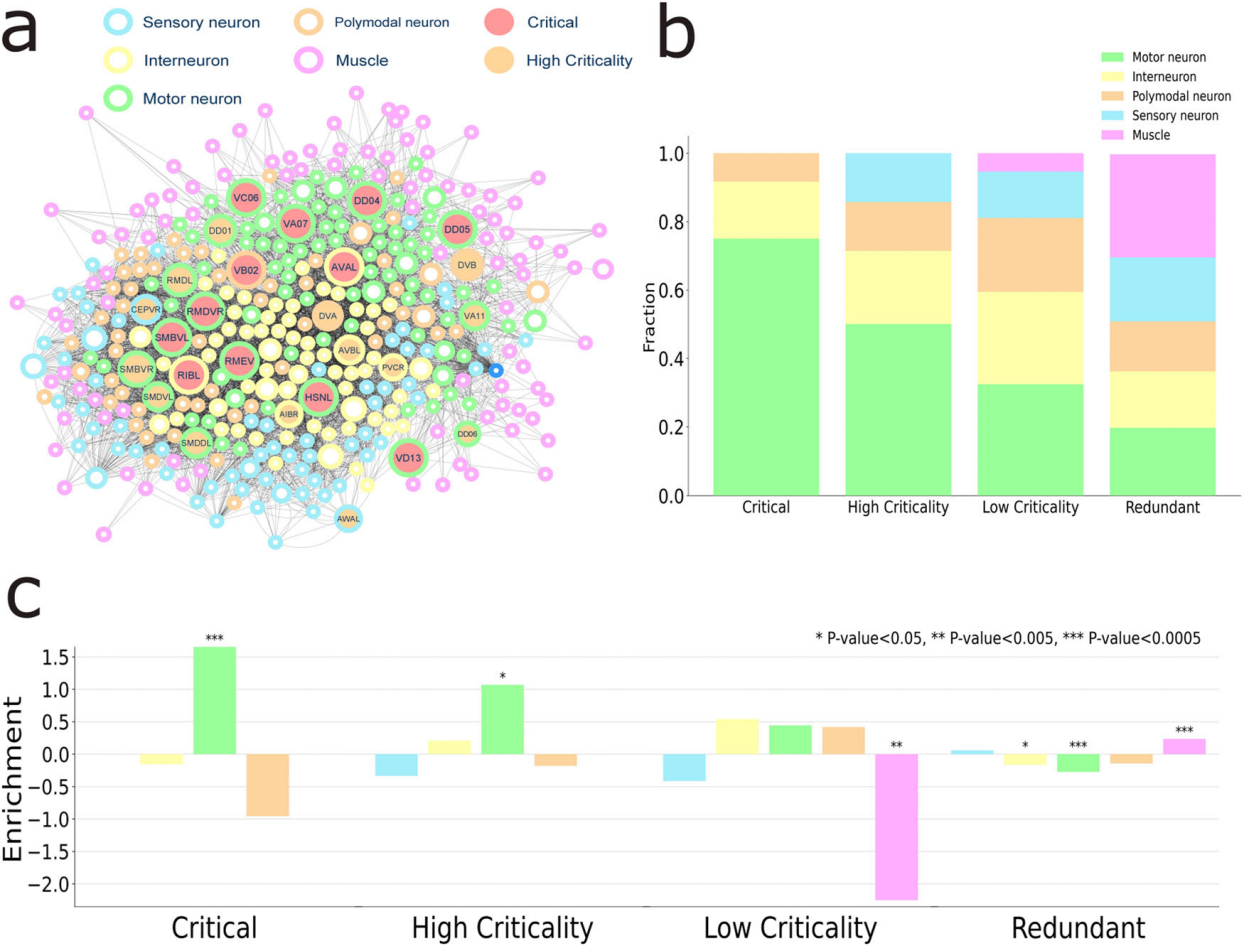
Criticality in the connectome network. **a** The *C. elegans* connectome network with circle colors denoting the neuron function according to figure legend and filled nodes referring to critical (red) and high criticality (orange) categories. **b** The fraction of neuron types in each control category. **c** Enrichment analysis for each control category and neuron class. All p-values derived from two-tailed Fisher’s exact tests.

By using this network, we applied the criticality algorithm and unveiled for the first time a subset of neurons that have a high criticality. See the Supplementary Note 2 and Supplementary Table 1 (Excel file) for a detailed list of the identified high criticality neurons in the *C. elegans* network. Figure 10a shows the *C. elegans* connectome network with circle colors denoting the neuron function according to figure legend and filled nodes referring to critical (red) and high criticality (orange) categories identified by the algorithm. Figure 10b shows the fraction of neuron types in each control category. The results show that the largest fraction of critical nodes as well as high criticality nodes are motor neurons. The amount of motor neurons in each control category decreases further from low criticality to redundant nodes. On the other hand, muscles play a more passive role in terms of control and therefore, they tend to appear in low criticality and redundant categories. High criticality nodes are also composed of sensory, interneuron and poly-modal neurons. No critical protein played a sensory role, however, intermittent neurons with high criticality may also be engaged as sensory tasks. The enrichment analysis shows the largest enrichment scores for critical and high criticality nodes for motor neurons (*p*-value = 0.0002 and *p*-value = 0.047; two-tailed Fisher’s exact tests, respectively) (see Fig. 10c). These results show not only reasonable functional findings explained by the MDS control approach but also demonstrate that the uncovered intermittent nodes with high criticality play more important and variate biological roles than previously thought.

Here we also compared our results with those obtained from the set of high degree (Supplementary Fig. 7a, c) and the set of top degree nodes (Supplementary Fig. 7b, d). See also Supplementary Note 3. First, the analysis of the fraction of nodes (Supplementary Fig. 7a, b) indicates that high criticality nodes target a much more diverse set of nodes. While for the set of high degree nodes motor neurons are still identified in a low percentage (Supplementary Fig. 7a), they are completely overlooked for the set of top degree nodes (Supplementary Fig. 7b). Regarding the enrichment analysis, we observed similar findings. For the sets of high degree nodes (Supplementary Fig. 7c) and top degree nodes (Supplementary Fig. 7d), the out-degree nodes tend to target interneurons, while the high criticality nodes target motor neurons. These results confirm that the high criticality metric extracts unique information from the network, that could not be derived by using degree-based metrics.

## Discussion

In this work, we have proposed an efficient algorithm based on a new criticality metric to analyse and determine the functional roles of intermittent nodes in complex biological networks. The computation of the criticality metric in a large network is challenging because by increasing the network size, the number of solutions of the MDS increases very fast. The criticality definition requires knowing all the possible MDS solutions of a given network, therefore it was necessary to develop an efficient algorithm to compute this metric. The algorithm can be applied to any type of directed networks. In this work, the application of this algorithm to several directed networks unveiled the importance and functional roles of intermittent nodes in different systems, from cellular level such as signaling pathways and cell-cell interactions such as cytokine networks, to large scale nervous systems from *C. elegans*. In our view, the proposed criticality metric and its efficient computation algorithm may open new avenues for investigating the role of intermittent nodes in many biological systems of interest in the context of the MDS framework for network control.

## Methods

### Hamming distance between minimum dominating sets

From the definition in Eq. 1, the calculation of criticality requires the computation of all MDS solutions. However, the number of the solutions of the MDS increases at a very fast rate as the network size increases, making it very difficult to calculate it in a large-scale network. In order to efficiently and approximately calculate the criticality in large networks, we introduce the concept of Hamming distance between MDSs in the proposed algorithm.

Let $G\left(V,E\right)$ be a graph with a set of nodes $V=\left\{{v}_{1},{v}_{2},\ldots ,{v}_{N}\right\}$ and a set of edges $E=\left\{{e}_{1},{e}_{2},\ldots ,{e}_{M}\right\}$. For a binary vector **b,** **b**[*i*] denotes the *i*th element of **b**. We represent a subset *S* of *V* by an *n*-dimensional binary vector **b**_*S*_ such that **b**_S_[*i*] = 1 iff. ${v}_{i}\in {\rm{S}}$. Let ${d}_{H}\left({{\bf{b}}}_{1},{{\bf{b}}}_{2}\right)$ denote the Hamming distance between two binary vectors **b**_1_ and **b**_2_. Then, we can obtain the following two propositions. Note that, intuitively, **bs** represents an MDS and that the Hamming distance between **b**_1_ and **b**_2_ represents how many nodes are different or swapped between two MDSs.

**Proposition 1**

Suppose that both *S*_1_ and *S*_2_ are minimum dominating sets of $G\left(V,E\right)$. Then, ${d}_{H}\left({{\bf{b}}}_{{S}_{1}}{\boldsymbol{,}}{{\bf{b}}}_{{S}_{2}}\right)$ is even.

(Proof)

Let *MDS* be a minimum dominating (MDS) for $G\left(V,E\right)$. Let ${S}_{1}\backslash {S}_{2}=\left\{s,|,s\in {S}_{1},s\notin {S}_{2}\right\}.$ We define the following numbers:

- $${x}_{1}=\left|{S}_{1}{\rm{\backslash }}{S}_{2}\right|$$,
- $${x}_{2}=\left|{S}_{2}{\rm{\backslash }}{S}_{1}\right|$$,
- $$y=\left|{S}_{1}\cap {S}_{2}\right|$$.

Since both *S*_1_ and *S*_2_ are MDSs, we have ${x}_{1}+y={x}_{2}+y={|MDS|}$ from which ${x}_{1}={x}_{2}$ follows. Therefore, the proposition follows from the following equality:

2 $${d}_{H}\left({{\bf{b}}}_{{S}_{1}},{{\bf{b}}}_{{S}_{2}}\right)={x}_{1}+{x}_{2}=2{x}_{1}=2{x}_{2}$$

**Proposition 2**

Let *MDS*, *CMDS*, and *IMDS* be a minimum dominating set, the set of critical

nodes, and the set of intermittent nodes, respectively. Then,

3 $${d}_{H}\left({{\bf{b}}}_{{S}_{1}},{{\bf{b}}}_{{S}_{2}}\right)\le 2\cdot \min \left(\left|{IMDS}\right|+\left|{CMDS}\right|-\left|{MDS}\right|,\left|{MDS}\right|-\left|{CMDS}\right|\right).$$

Let *S*_1_ and *S*_2_ be MDSs. We define *x* and *y* by

4 $$x=\left|{S}_{1}{\rm{\backslash }}{(S}_{1}\cap {S}_{2})\right|={\rm{|}}{S}_{2}{\rm{\backslash }}{(S}_{1}\cap {S}_{2}){\rm{|}}$$

5 $$y={\rm{|}}{(S}_{1}\cap {S}_{2}){\rm{\backslash }}{CMDS}{\rm{|}}.$$

Then, we have the following:

6 $$x+y+{|CMDS|}={|MDS|},$$

7 $${d}_{H}\left({{\bf{b}}}_{{S}_{1}},{{\bf{b}}}_{{S}_{2}}\right)=2x,$$

8 $$2x+y\le |IMDS|,$$

where the last inequality comes from the following facts:

9 $${\rm{|}}({S}_{1}\cup {S}_{2}){\rm{|}}=2x+y+{\rm{|}}{CMDS}{\rm{|}},$$

10 $$({S}_{1}\cup {S}_{2}){\rm{\backslash }}{CMDS}\subseteq {IMDS}.$$

We can see from Eqs. 6 and 8 that the following hold:

11 $$2x\le {\rm{|}}{IMDS}{\rm{|}}-y={\rm{|}}{IMDS}{\rm{|}}-{\rm{|}}{MDS}{\rm{|}}+{\rm{|}}{CMDS}{\rm{|}}+x,$$

12 $$x\le {\rm{|}}{IMDS}{\rm{|}}-{\rm{|}}{MDS}{\rm{|}}+{\rm{|}}{CMDS}{\rm{|}}.$$

Therefore, we have:

13 $${d}_{H}\left({{\bf{b}}}_{{S}_{1}},{{\bf{b}}}_{{S}_{2}}\right)=2x\le 2\left(\left|{IMDS}\right|+\left|{CMDS}\right|-\left|{MDS}\right|\right).$$

In addition, we can see from Eq. (6) and y ≥ 0 that the following holds:

14 $$x\le {\rm{|}}{IMDS}{\rm{|}}-{\rm{|}}{CMDS}{\rm{|}}.$$

Therefore, we have:

15 $${d}_{H}\left({{\bf{b}}}_{{S}_{1}},{{\bf{b}}}_{{S}_{2}}\right)=2x\le 2\left(\left|{IMDS}\right|-\left|{CMDS}\right|\right)$$

### Efficient algorithm for computing criticality in networks

Here we present an algorithm for the computation of the newly proposed criticality control category metric (See also Fig. 1d for a simplified schema of the algorithm steps). The algorithm uses the Hamming distance as well as the two mathematical propositions presented in the above section. The algorithm is implemented by following the steps below:

1. Determine all nodes in the network into the MDS control categories (critical, intermittent and redundant) using the Critical MDS (CMDS) control category classification algorithm and select one MDS, which is called *S*_1_.
2. Set constraints *x*_*i*_ = 1 and *x*_*i*_ = 0 on the critical and redundant vertices determined from step (1), respectively.
3. Calculate the maximum difference *n* between the MDS solution (*S*_1_) and the newly identified MDS solution (*S*_2_) in this step, and the initial value of the Hamming distance *K* for the ILP computation by using the network control configuration obtained from step (1).
4. Set the constraint that the Hamming distance (between an existing MDS solution and the newly identified MDS solution) must be greater or equal than *K*
5. Calculate an MDS solution by using ILP computation under the constraints of step (4) (see Eq. 20).
6. Repeat steps 4 and 5 until there are no more MDS solutions.
7. Calculate the criticality metric as shown in Eq. 1.
8. Compute the difference of the criticality between the current and previous *K* calculation. (Note this step can only execute from the second round since we need two criticality values to compute the difference).
9. Update the constraints on the Hamming distance (see details below).
10. Repeat the steps (4 to 9) until the specific conditions for algorithm termination are satisfied (see details below).

The constraints on the Hamming distance for the identified MDS solution in step (3) are explained as follows. If the identified MDS solution at step (1) is *S*_1_ and the new arbitrarily obtained MDS solution in step (3) is *S*_2_, the maximum difference *n* between these two MDS solutions can be calculated as follows:

Let us consider $h=\left|{S}_{2}\backslash {S}_{1}\right|$, then the Hamming distance ${d}_{H}\left({{\bf{b}}}_{{S}_{1}},{{\bf{b}}}_{{S}_{2}}\right)$ between *S*_1_ and *S*_2_ can be expressed as

16 $${d}_{H}\left({{\bf{b}}}_{{S}_{1}},{{\bf{b}}}_{{S}_{2}}\right)=2h\le 2\min \left(\left|{IMDS}\right|+\left|{CMDS}\right|-\left|{MDS}\right|,\left|{MDS}\right|-\left|{CMDS}\right|\right).$$

By invoking Proposition 1 and 2, the above expression can be rewritten in terms of *h* and reads as

17 $$h=\frac{{d}_{H}\left({{\bf{b}}}_{{S}_{1}},{{\bf{b}}}_{{S}_{2}}\right)}{2}\le \min \left(\left|{IMDS}\right|+\left|{CMDS}\right|-\left|{MDS}\right|,\left|{MDS}\right|-\left|{CMDS}\right|\right).$$

Therefore, when $h=\min \left(\left|{IMDS}\right|+\left|{CMDS}\right|-\left|{MDS}\right|,\left|{MDS}\right|-\left|{CMDS}\right|\right)$, the maximum value is obtained, which can be used to define *n*.

Next, we explain how to determine the Hamming distance *K* for the ILP calculation. Let us consider ${x}_{i}\left({x}_{i}\in {S}_{1}\cap {S}_{2}\right)=-1$, ${x}_{i}\left({x}_{i}\in {S}_{2}\backslash {S}_{1}\right)=1$, ${x}_{i}\left({x}_{i}\in \bar{{S}_{2}}\right)=0$, and $h=\left|{S}_{2}\backslash {S}_{1}\right|$, then we define the Hamming distance *K* for the ILP calculation as the sum of *x*_*i*_ in *S*_2_, and that can be written as

18 $$K=-\left|{CMDS}\right|-\left(\left|{MDS}\right|-\left|{CMDS}\right|-k\right)+h=2h-\left|{MDS}\right|.$$

By using the obtained *n* (as shown above), we can calculate the initial value of the Hamming distance *K* for the ILP calculation, that is

19 $$K=2n-\left|{MDS}\right|.$$

Then, by using the *K* obtained above, the ILP formulation in step (4) is given by the following Eq. 20.

**Minimize**

$$\sum _{v\in V}{x}_{v}$$

**Subject to**

20 $$\begin{array}{c}{x}_{u}+\sum _{(v,u)\in E}{x}_{v}\ge 1\, \forall u\in V,\\ {x}_{v}=1\,\forall v\in CMDS,\\ {x}_{v}=0\,\forall v\in RMDS,\\ -\sum _{v\in S}{x}_{v}+\sum _{v\in \bar{S}}{x}_{v}\ge K\,\forall S\in MDSs,\\ {x}_{v}\in \{0,1\}\,\forall v\in V.\end{array}$$

In addition, as the *MDSs* include the solutions of all calculated MDS, the constraint equation related to Hamming distance *K* (see Eq. 20) requires ${\rm{|}}{MDSs|}$ equations. Note that the number of these equations increases proportionally to the number of MDS solutions.

By updating the constraint equation in step (9), the definition of the Hamming distance *K* ($K=2h-\left|{MDS}\right|$) for the ILP calculation indicates that if $h=\left|{S}_{2}\backslash {S}_{1}\right|$ changes by 1, the Hamming distance *K* changes by 2, therefore the Hamming distance *K* is updated as *K* = *K*-2 for each round (see Eq. 20).

The computation to determine the difference in criticality mentioned in step (8) is explained below.

When $\left|{IMDS}\right|=I$, the criticality of *v*_*i*_ computed at the Hamming distance *K* is expressed as ${{CR}}_{i}^{K}$. Then, the difference of criticality ${{dCR}}_{K,K-2}$ between *K* and *K-2* is calculated by using Eq. 21.

21 $${{dCR}}_{K,K-2}=\sqrt{\frac{1}{I}\mathop{\sum }\limits_{i=1{\rm{;}}{v}_{i}\in {IMDS}}^{N}{\left({{CR}}_{i}^{K}-{{CR}}_{i}^{K-2}\right)}^{2}}$$

While the critical and redundant nodes have a criticality score of 1 and 0, respectively, the intermittent nodes are the only ones that may change their criticality score between *K* and *K-2*. Therefore, in Eq. 21, ${{dCR}}_{K,K-2}$ is computed using only intermittent nodes.

Regarding the specific conditions mentioned in step (10), the algorithm is terminated when ${{dCR}}_{K,K-2}$ is below the pre-defined termination threshold *θ*. The value of *θ* is a free parameter that can be set up in the algorithm before computation starts. By decreasing this value, we can increase the precision of the computed criticality.

When $\left|{IMDS}\right|\ne 0$ ($\left|{IMDS}\right|=0$) because the minimum value of $h=\left|{S}_{2}\backslash {S}_{1}\right|$ is 1 (0), from the definition of Hamming distance *K* ($K=2h-\left|{MDS}\right|$), the calculation is terminated when $K=2-\left|{MDS}\right|$ ($K=-\left|{MDS}\right|$), respectively. Therefore, by combining the above two cases, the termination condition can be written as $K\le 2-\left|{MDS}\right|$ (see Fig. 1d).

In this algorithm, the Hamming distance *K* takes its maximum value at the beginning of the computation. Therefore, it is possible to start the MDS calculation from the most constrained case where intermittent nodes in the MDS solutions must be interchanged. By gradually relaxing the constraint of the number of interchanges, it is possible to efficiently discriminate between nodes included in many MDS solutions (high criticality) and those included only in a few MDS solutions (low criticality).

### Evaluation methods for the high criticality proteins

#### Enrichment analysis

To investigate the relationship between high criticality (HCR) proteins and disease-related proteins, the enrichment score was computed. The enrichment score indicates how abundant these proteins are in the set of high criticality proteins. The computation method is described as follows:

First, the ratio *f*^*D*^ indicates the fraction between the number of the disease-associated proteins *N*^*D*^ and the total number of proteins *N* constituting the network:

22 $${f}^{D}=\frac{{N}^{D}}{N}$$

The ratio ${f}_{{HCR}}^{D}$ indicates the fractions between the number of each disease-related protein in the high criticality category ${N}_{{HCR}}^{D}$ and the number of high criticality proteins *N*_*HCR*_ in the network:

23 $${f}_{{HCR}}^{D}=\frac{{N}_{{HCR}}^{D}}{{N}_{{HCR}}}$$

From above equations, we can define the enrichment ${E}_{{HCR}}^{D}$ of a set of proteins (related to diseases or other biological function of interest in general) in the set of high criticality proteins as follows:

24 $${E}_{{HCR}}^{D}={\log }_{2}\left(\frac{{f}_{{HCR}}^{D}}{{f}^{D}}\right)$$

The statistical significance of these values was calculated by Fisher’s exact probability test (two-tailed test). In this study, the relationship between high criticality proteins and each disease-related protein is considered to be statistically significant when the p-value calculated by the test is less than 0.05.

The same method was used to calculate the enrichment of phosphorylation mechanisms-associated proteins as well as for the enrichment of each functional neuron category in the set of high criticality proteins in the *C. elegans* organism.

The enrichment for different control categories was also computed with this method replacing the set of high criticality by those of each control category such as critical, intermittent and redundant. Note that the analysis classified the intermittent set into high criticality and low critical sets based on the criticality score (see Eq. 1) above or below 0.5, respectively.

### Assessment of high criticality nodes in control of network module

In order to verify that the high criticality nodes have stronger control properties on the COVID inflammation module, the metrics described below were calculated. In each following equation, *S*_*M*_ represents the set nodes included in the module disease, and *S*_*HCR*_ represents the set of identified high criticality nodes.

25 $$< d > =\frac{1}{\left|{S}_{{HCR}}\right|\times \left|{S}_{M}\right|}\sum _{{v}_{j}\in {S}_{{HCR}}}\sum _{{v}_{i}\in {S}_{M}}d\left({v}_{j},{v}_{i}\right)$$

In Eq. 25, $d\left({v}_{j},{v}_{i}\right)$ represents the shortest path distance from nodes *v*_*j*_ to *v*_*i*_.

The shortest path distance means the minimum number of edges that must be passed from the initial node to the destination node. Therefore, <*d*> means the average of the sum of all shortest path distances from each high criticality node to each module node. Thus, from Eq. 8, the closer this value is to 1.0, the deeper the structural association between high criticality and the disease module is in the network.

Next, Eqs. 26 and 27 show the metrics for *links* and *cov*, respectively.

26 $${links}=\sum _{{v}_{i}\in {S}_{M}}\left|\left\{{v}_{j}\in {S}_{{HCR}}{\rm{|}}\left({v}_{j},{v}_{i}\right)\in E\right\}\right|$$

27 $$\mathrm{cov}=\frac{1}{\left|{S}_{M}\right|}\sum _{{v}_{i}\in {S}_{M}}\min \left(1,\left|\left\{{v}_{j}\in {S}_{{HCR}}{\rm{|}}\left({v}_{j},{v}_{i}\right)\in E\right\}\right|\right)$$

where *E* indicates the set of directed edges.

The *links* means the total number of all directed edges outgoing from any high criticality node and incoming to any node in the module. The *cov* means the fraction of nodes in the module that have an incoming edge from high criticality nodes (that is, the module coverage ratio by the high critical nodes). From both equations, the higher the value of *links* indicates that the high criticality nodes have a greater impact on the module, and the closer the value of *cov* is to 1.0, the more effective the control of the high criticality nodes is in controlling the entire module.

After calculating the metric scores for the high criticality nodes (red arrows in Fig. 9), to assess the randomness impact and to evaluate the statistical significance of the results, we performed a statistical analysis as follows. We constructed sets of nodes with same size of the number of observed high criticality nodes. Each set of nodes was randomly selected from the entire network and their metric values were calculated. This random selection was performed 10,000 times. The results are shown (see Fig. 9) as histogram plots of the distribution of the calculated metric values in the random samples and a comparison with the metric values of the observed high criticality nodes (red arrows).

### Network centrality metrics

To compute the closeness, betweenness and Page Rank network metrics we used the Networkx python package version: 2.8.4.

### Reporting summary

Further information on research design is available in the Nature Research Reporting Summary linked to this article.

### Supplementary information

Supplementary Material

Supplementary Table 1

Reporting summary

## Data Availability

The data used in this study is publicly available from databases^25,35^ and from previous publications (Supplemental Material section) referenced in the text^8,29,33^: 10.1073/pnas.1603992113^8^, 10.1126/science.1257601^29^, 10.3390/cells10092242^33^. The list of the identified intermittent proteins and neurons nodes with high criticality is shown in the Supplementary Table 1 (Excel file) that accompanies this manuscript. Data that support the tables and figures of this study are available from the corresponding author upon request.

## Code availability

The custom code for the developed algorithm used in the analysis is publicly available from https://github.com/wataru-s1/criticality.
