# Supplementary material for: Measuring criticality in control of complex biological networks
Source: NPJ Syst Biol Appl. 2024 Jan 20;10:9. doi: 10.1038/s41540-024-00333-9 (PMC10799883; doi:10.1038/s41540-024-00333-9)
Supplement: Supplementary file 1 — Supplementary Material [file 41540_2024_333_MOESM1_ESM.pdf]

Supplementary Information:
Measuring criticality in control of complex biological networks

Wataru Someya[1], Tatsuya Akutsu[2],
Jean-Marc Schwartz[3] and Jose C. Nacher[1]

[1] *Department of Information Science, Faculty of Science, Toho University, Funabashi, Chiba 274-8510, Japan*

[2] *Bioinformatics Center, Institute for Chemical Research, Kyoto University, Uji, 611-0011, Japan*

[3] *School of Biological Sciences, University of Manchester, Manchester M13 9PT, UK.*

Background

Controllability methods integrates network science with control theory concepts, and have shown promising results identifying biological molecules that not only play a crucial role in the network control, but also are associated with specific disease and cellular functions. In both leading proposed approaches, namely, maximum matching[1] and minimum dominating sets (MDS) [2] , the algorithmic solutions are not unique, and nodes are classified as critical (if one node belongs to all solutions, intermittent (if at least they belong to 1 or more solutions but not all), and redundant (if a node does not belong to any solution). While most research has focused on the importance of critical nodes [3], the intermittent nodes have received much less attention, in spite of the fact they are also engaged in network control.

Here, we propose mathematical and algorithmic tools to study the role of intermittent nodes in biological networks and use a criticality metric (see Eq. 1 in main text) to quantify its importance. This control metric measures the relevance of each intermittent node based on the number of times it appears in an MDS solution. It is well-known that the MDS is an NP-hard problem, therefore, its computation is challenging. Indeed, although the criticality is a simple concept, its computation is far from easy in large networks, and requires a sophisticated algorithm to be computed in feasible time. In particular, we derived the following mathematical propositions that significantly enhanced the performance of the algorithm in large networks (see Methods section in main text). In what follows, the Supplementary Note 1 includes additional computational results on the assessment of the algorithm results. The Supplementary Note 2 describes the excel file that includes the list of high criticality proteins identified in the RTK signaling pathway and in the *C. elegans* neural network. The Supplementary Note 3 includes additional results using different centrality metrics for the analysis of the cytokine network and the *C. elegans* neural network.

Supplementary Note 1: Assessment on the algorithm results

To assess that the heuristic algorithm gives results that are very close to the actual values, we performed the following computations using artificially generated scale-free networks of N=200 (Supplementary Fig. 1a) and N=300 (Supplementary Fig. 1b) nodes. As shown in main text, the criticality of node v_i at the Hamming distance K is denoted as CR_i^K. Then, by considering $|IMDS|=I$, the criticality difference between two Hamming distances (dCR) is expressed as Eq. 21 (see main text). Similarly, we can write the error between the criticality computed at each Hamming distance K and the real (exact) observed criticality (denoted as CR_i^R) as follows:

$$Error = \sqrt{\frac{1}{I}\sum_{i=1;v_i \in IMDS}^{N}(CR_i^K - CR_i^R)^2} \quad (1)$$

The results are shown in Supplementary Fig. 1, and indicate that when the criticality difference between two Hamming distances decreases, the error also decreases. The computation terminates when the dCR reaches the predetermined threshold θ. Supplementary Fig. 1 shows that the error is very small, and that it decreases slightly more by decreasing θ from 0.02 and 0.01.

And additional test was done by plotting the computation of criticality value CR_i^K at threshold θ=0.01 versus the actual (exact) criticality value CR_i^R for each node v_i (see Supplementary Fig. 2ab). As shown in figure, the computed criticalitiy and the exact value show a diagonal pattern indicating the correctness of the computation.

To verify that the algorithm can obtain very close results to the actual values using scale-free networks generated using different parameters such as network size, average degree and degree exponent, we performed additional computational experiments. Moreover, experiments were also done to compare the results obtained in scale-free networks with those from different network structures such as Erdős Rényi (ER) (random graphs).

Supplementary Fig. 3a-b show the results for scale-free networks with $N = 200$ and $<k>$=2, and γ=2.2, 3.0. Note that Supplementary Fig. 1a and Supplementary Fig. 2a show results for scale-free networks for N=200 with $<k>$=3 and γ=2.5. The results show that there are no significant differences when structural parameters are changed, and the error between the computed and observed criticality remains small.

Supplementary Fig. 4a-d display the results for scale-free networks with $N = 300$, and several combinations of average degree $<k>$=2, 5 and degree exponent γ=2.2, 3.0. Note that Supplementary Fig. 1b and Supplementary Fig. 2b also show the case for N=300 with $<k>$=3 and γ=2.5. Again, the increment of network size and the changes of the structural parameters do not significantly modifiy the results and the error is still small.

The computations done using Erdős Rényi networks with N=200 and N=300 nodes are compared with those of scale-free networks of the same size (see Supplementary Fig. 3c and Supplementary Fig. 4e, respectively). The results do not show noticeable changes when the network structure is drastically changed from scale-free networks to random networks.

Finally, by increasing the network size up to N=400 nodes and by using several scale-free network configurations of average degree and degree exponent, the error does not seem to change and still remains small (see Supplementary Fig. 5a-b).

To sum up, the performed computational experiments show that the algorithm is reliable to offer results that are very close to the actual values in random and scale-free networks

of moderate size of a few hundreds nodes. We also expect that the proposed algorithm can stimulate research on this topic, and may lead to further develop the ideas and techniques introduced in our analysis.

Supplementary Note 2: List of the identified high criticality proteins and neurons (Supplementary Table 1 (Excel file))

A Supplementary Table 1 (excel file) accompanies the manuscript with a list of the identified high criticality proteins in the RTK signaling pathway. The file includes the results for the analysed biological datasets, including the human diseases. For each protein, the Uniprot ID, gene symbol and Gene ID are shown (see Sheet1). In the same file, the neurons of *C. elegans* organism with high criticality score are also listed. The second column denotes the neuron functionality (see Sheet 2).

Supplementary Note 3: Additional degree-based centrality metrics results on the cytokine interaction network and C. elegans neural network

The results shown in Fig. 9 (main text) demonstrate that the high criticality nodes (the four nodes shown in yellow in Fig 8a) identified in the cytokine interaction network have stronger control properties on the COVID inflammation module than randomly selected nodes. To assess our results in comparison with other metrics, we selected five centrality metrics, namely, closeness, betweenness, page rank, out-degree and total degree. We then computed the same metrics $< d >$, *links*, and *cov* by using the set of top degree/centrality nodes (Fig. 9) and the set of high degree/centrality nodes (Supplementary Fig. 6) ordered according to each degree/centrality measure mentioned above. The top degree/centrality set of nodes shows the best performance for the cov (coverage) of both the COVID module and the entire network Fig. 9cd), and shows the second rank in the results for $< d >$, and *link* measures (Fig. 9ab). Similarly, for the set of high degree/centrality nodes, the high criticality performs better than all the examined centralities for the coverage metric of the entire network (Supplementary Fig. 6d) and also gives the best result for the coverage of the COVID module with same score as the out-degree (Supplementary Fig. 6c). Regarding, $< d >$ and *links* metrics, high criticality scores second after the out-degree (Supplementary Fig. 6ab).

It is worth mentioning that coverage is the metric most closely related to actual control among the other two. Moreover, it is to be noted that the high criticality nodes are part of a set that satisfied controllability properties derived by using advanced techniques [2], while sets obtained by other centrality metrics such as pager rank or degree, although can be used to rank network nodes, do not satisfy controllability conditions.

Regarding the *C.elegans* network, we also compared our results on the high criticality metric with those obtained from the set of high degree (Supplementary Fig. 7ac) and the set of top degree nodes (Supplementary Fig. 7bd). First, the analysis of the fraction of nodes (Supplementary Fig. 7ab) indicates that high criticality nodes target a much more diverse set of nodes. While for the set of high degree nodes, motor neurons are still identified in a low percentage (Supplementary Fig. 7a), they are completely overlooked for the set of top degree nodes (Supplementary Fig. 7b). Regarding the enrichment analysis, we observed

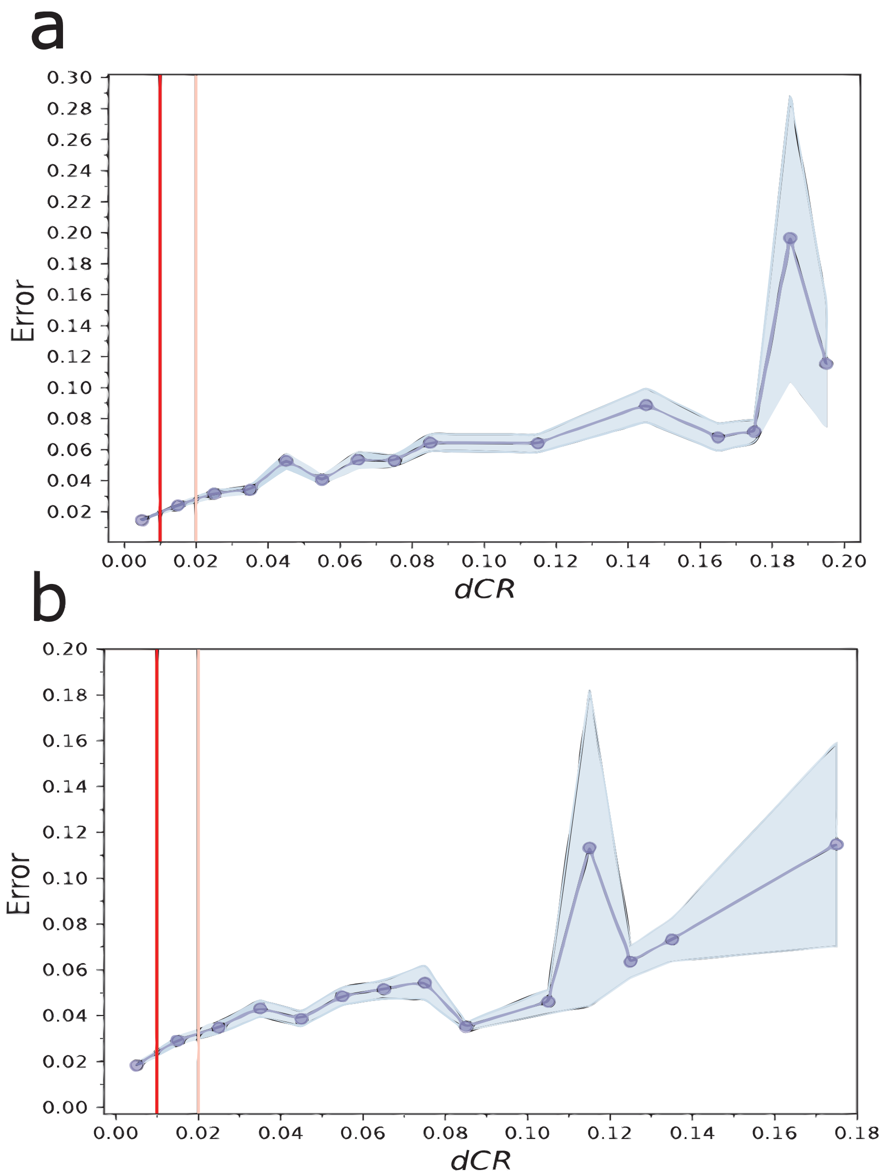

Supplementary Figure 1: The error of the results at Hamming distance K (Eq. 1 in Supplementary Note 1) versus the criticality difference between two Hamming distances (dCR) expressed as Eq. 21 in the main text. The results correspond to scale-free networks of size N=200 (a) and N=300 (b) with $< k >$=3 and γ=2.5, and averaged over ten network samples. The light blue overlay denotes the standard error of the results, which also decreases with Hamming distance. The termination threshold θ is denoted by continuous red line (0.01) and soft pink line (0.02).

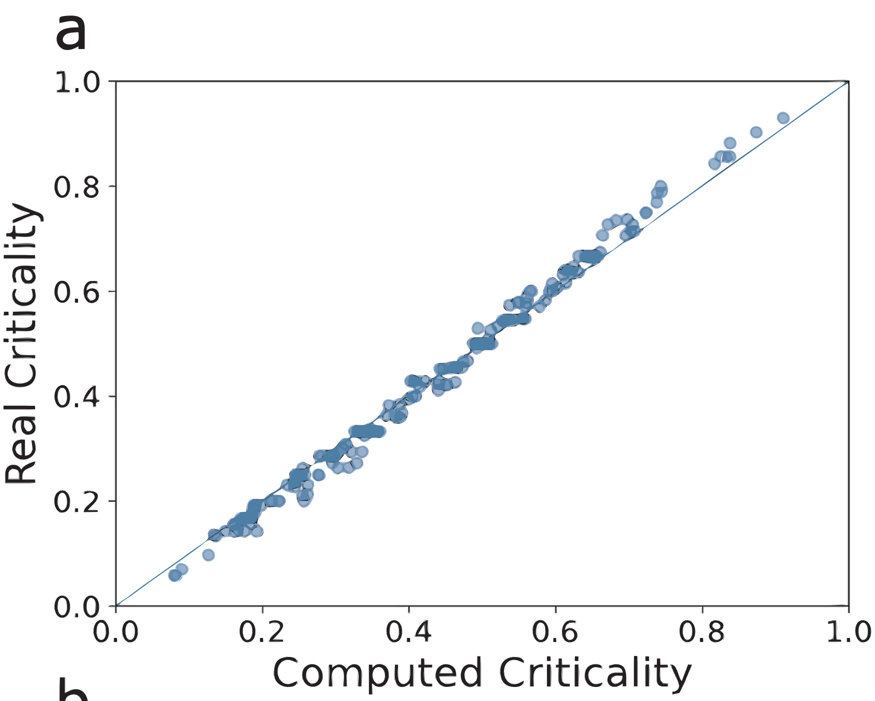

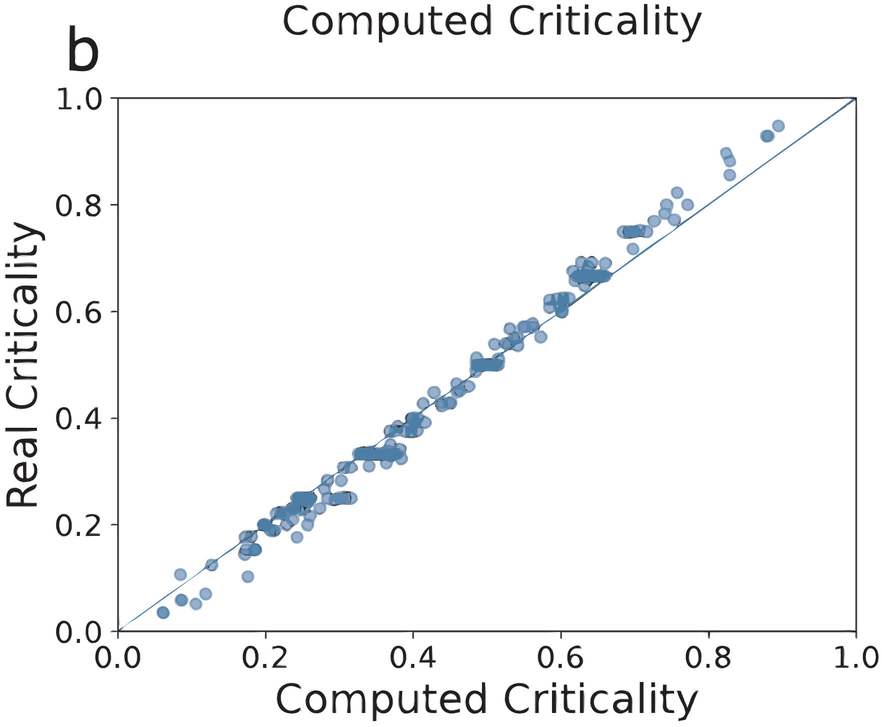

Supplementary Figure 2: The computation of criticality value CR_i^K at threshold $\theta = 0.01$ versus the real (exact) criticality value CR_i^R for each node v_i. (a) and (b) denotes the same network data as shown in Supplementary Fig. 1. From a total of (a) 2000 data points (nodes) and (b) 3000 data points (nodes), we plotted results for intermittent nodes. That is, we did not plot the redundant and critical nodes (i.e., criticality 0 or 1, respectively). In both experiments, the data results follow a diagonal indicating the correctness of the solution of the proposed criticality algorithm.

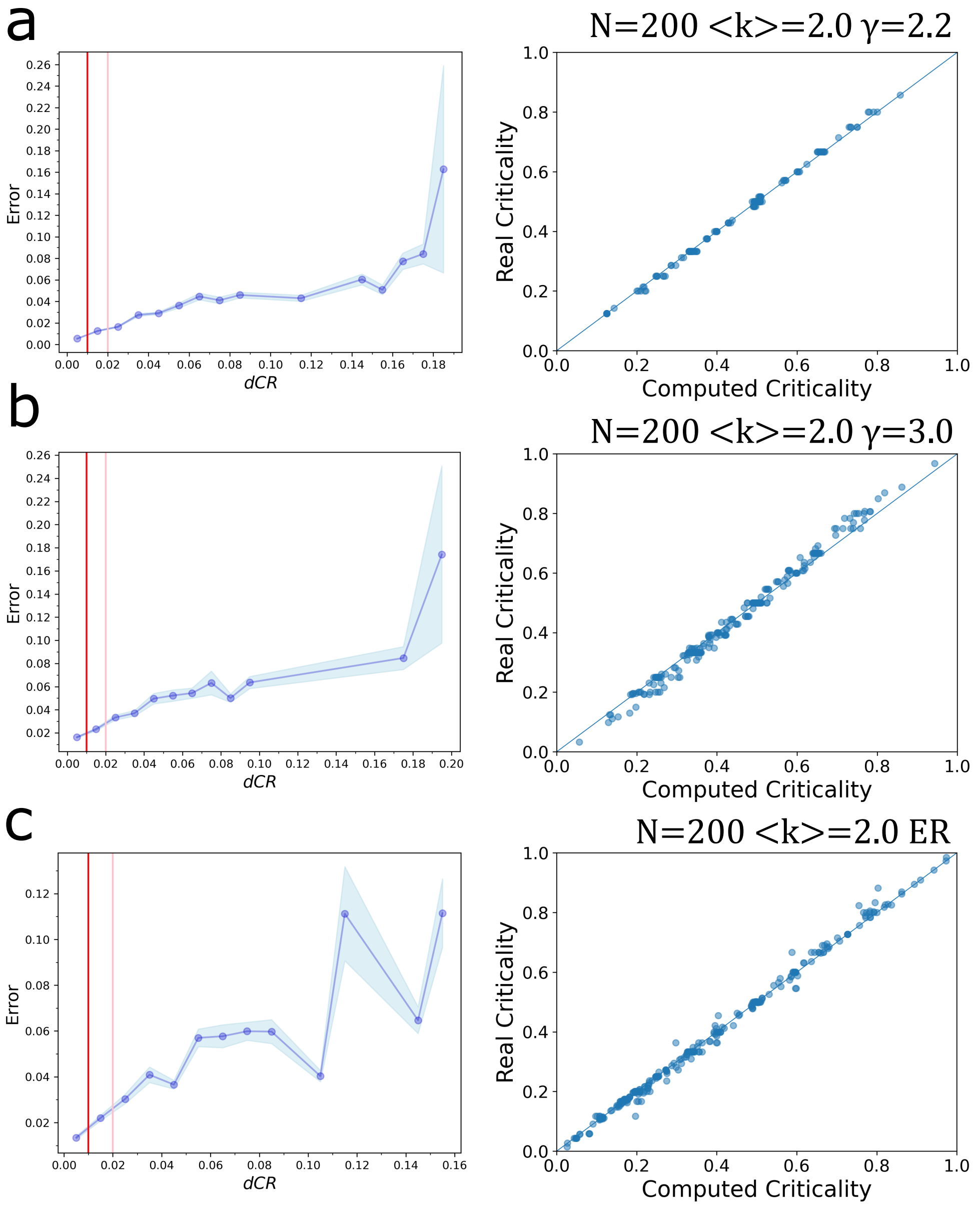

Supplementary Figure 3: (Left (a-b)) Same as Fig. 1a for scale-free networks with N=200 nodes and using the network parameters indicated in each panel. The results are averaged over ten network samples. The termination threshold θ is denoted by continuous red line (0.01) and soft pink line (0.02). (Right (a-b)) Same as Fig. 2a. The data results follow a diagonal indicating the correctness of the solution of the proposed criticality algorithm. (c) The results for Erdős Rényi networks. The results are also averaged over ten network samples.

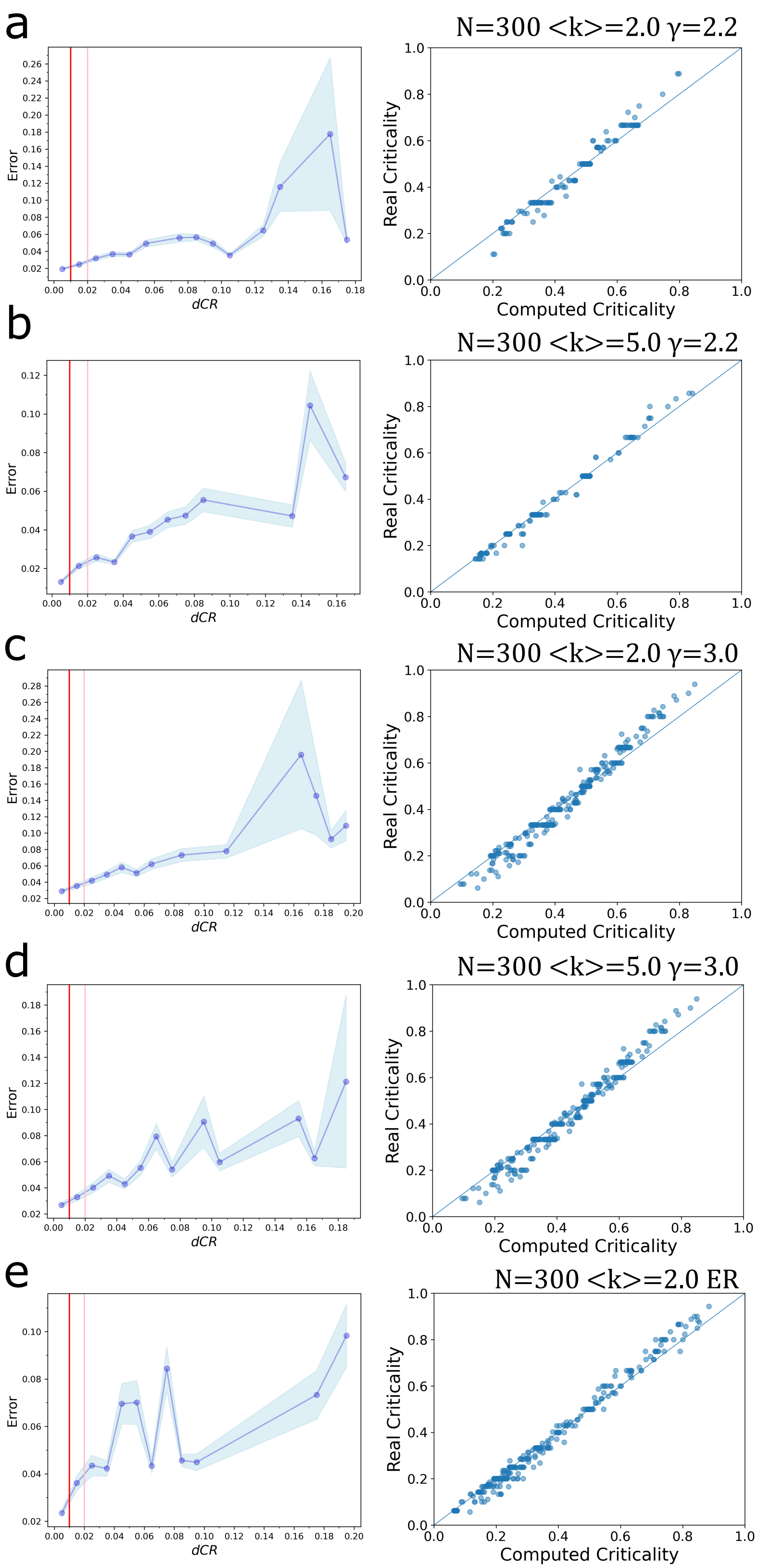

Supplementary Figure 4: (Left (a-d)) Same as Fig. 1a for scale-free networks with N=300 nodes and using the network parameters indicated in each panel. The results are averaged over ten network samples. The termination threshold θ is denoted by continuous red line (0.01) and soft pink line (0.02). (Right (a-d)) Same as Fig. 2a. The data results follow a diagonal indicating the correctness of the solution of the proposed criticality algorithm. (e) The results for Erdős Rényi networks. The results are also averaged over ten network samples.

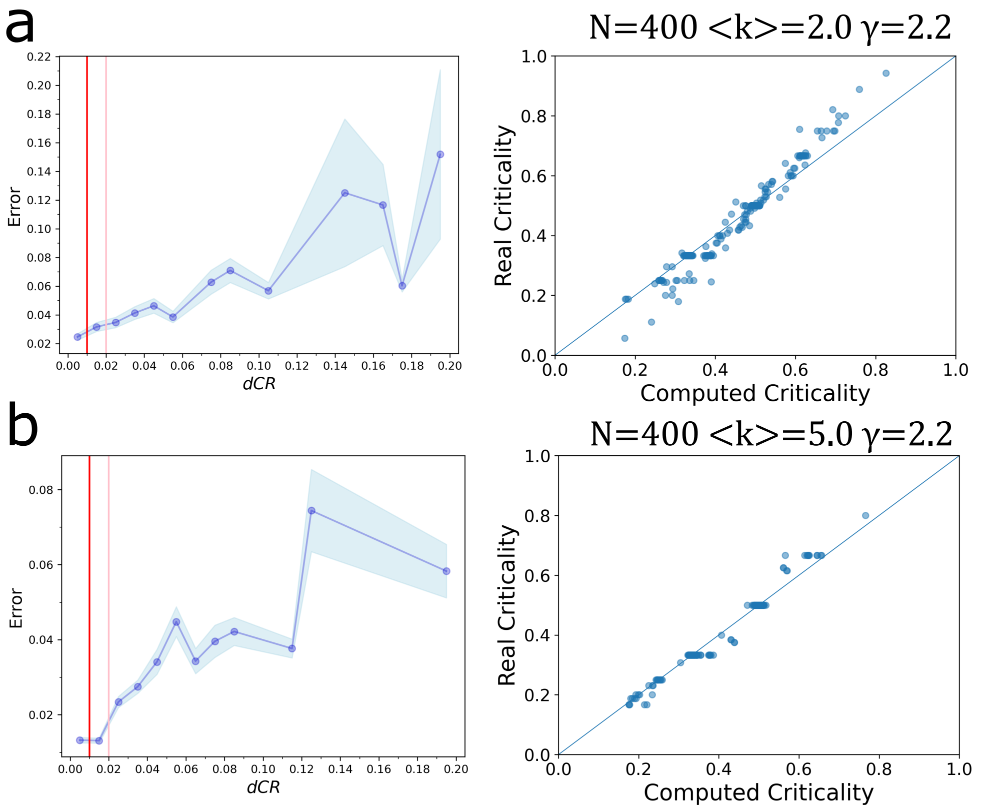

Supplementary Figure 5: (Left a-b)) Same as Fig. 1a but for for scale-free networks with N=400 nodes and using the network parameters indicated in each panel. The results are averaged over ten network samples. The termination threshold θ is denoted by continuous red line (0.01) and soft pink line (0.02). (Right (a-b)) Same as Fig. 2a. The data results follow a diagonal indicating the correctness of the solution of the proposed criticality algorithm.

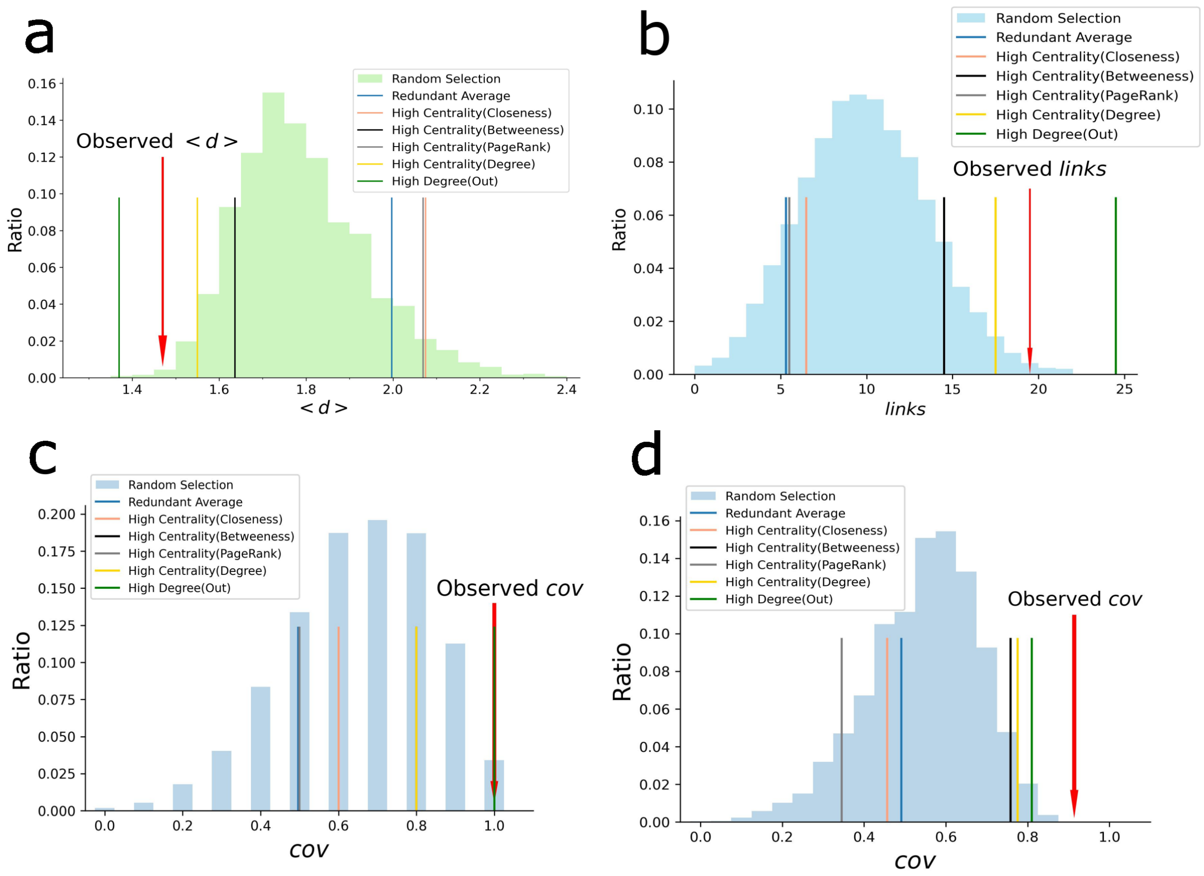

Supplementary Figure 6: (a-c) Histograms showing a comparison of $< d >$, *links* and *cov* metrics computed for the COVID-19 module in the cytokine network for high criticality nodes (red arrows) and randomly selected nodes (histogram bars). (d) The high criticality nodes coverage for the entire cytokine interaction network. The number of trials of random selection for each feature is 10,000. Each figure also displays the results for the high degree/centrality set of nodes computed using closeness, betweenness, page rank, out-degree and total degree metrics for comparison purposes.

similar findings. For the sets of high degree nodes (Supplementary Fig. 7c) and top degree nodes (Supplementary Fig. 7d), the out-degree nodes tend to target interneurons, while the high criticality nodes target motor neurons. These results confirm that high criticality metric extracts unique information from the network that could not be derived by using simpler degree-based metrics.

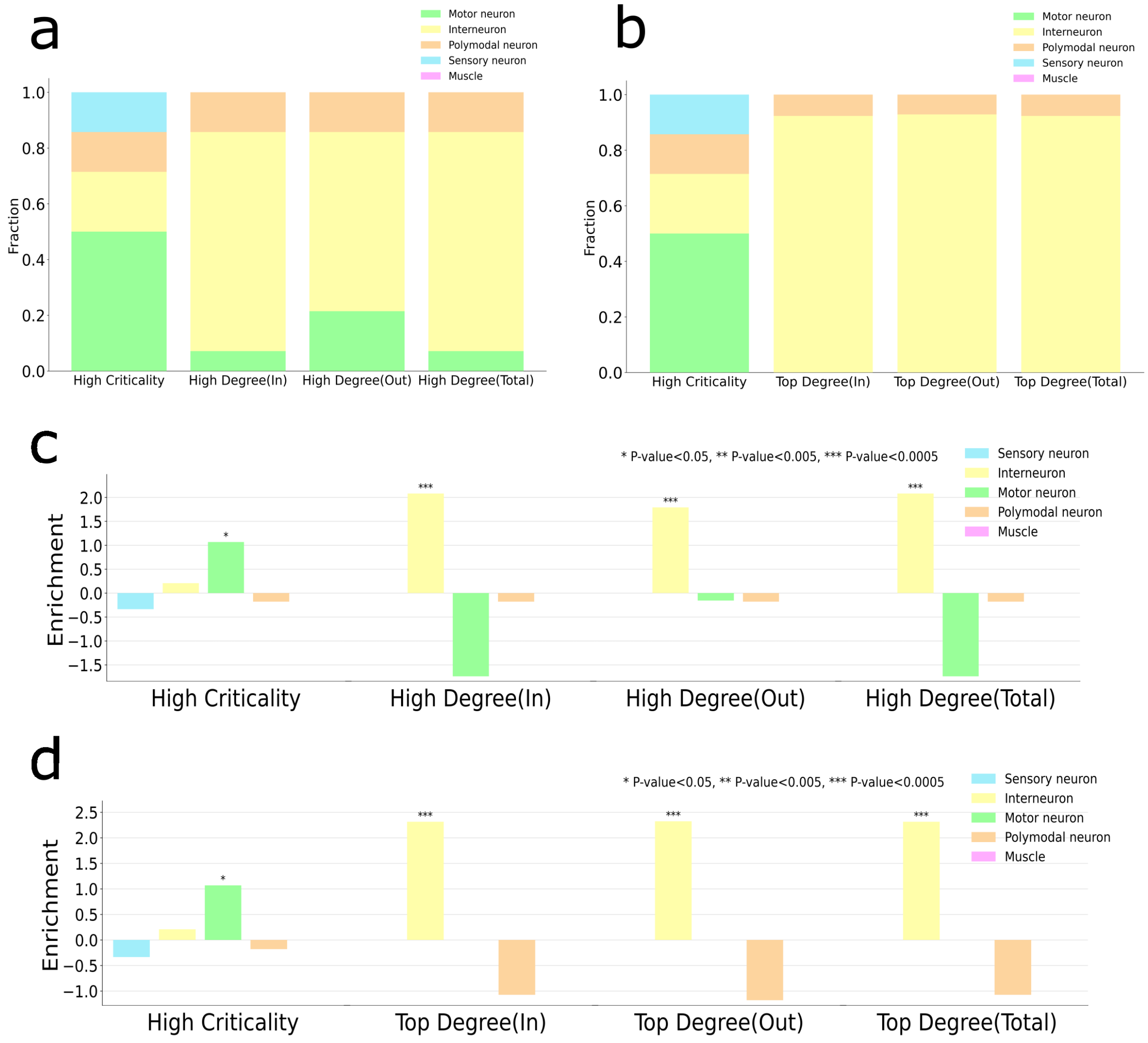

Supplementary Figure 7: (a) The fraction of neuron types in each control category computed using the set of high degree nodes and (b) the set of top degree nodes. (c) Enrichment analysis for each control category and neuron classes computed using the set of high degree nodes and (d) the set of top degree nodes. In all figures, the left hand side histogram shows the results for high criticality nodes for comparison purposes. All p-values derived from two-tailed Fisher's exact tests.

Supplementary References

[1] Liu, Y.-Y., Slotine, J.-J. & Barabási, A.-L. Controllability of complex networks. *Nature* **473**, 167-173 (2011).

[2] Nacher, J.C. & Akutsu, T. Dominating scale-free networks with variable scaling exponent: heterogeneous networks are not difficult to control. *New Journal of Physics* **14**, 073005 (2012).

[3] Kagami, H, Akutsu, T., Maegawa, S., Hosokawa, H. & Nacher, J.C. Determining associations between human diseases and non-coding RNAs with critical roles in network control. *Scientific Reports*, **5**:14577 (2015).
